# Albumin-Bound Paclitaxel (SYHX2011) in Patients with Advanced Breast Cancer: A Multicenter, Randomized, Double-Blind, Phase III Study

**DOI:** 10.34133/cancomm.0037

**Published:** 2026-06-26

**Authors:** Lina Zhang, Jingxuan Wang, Tao Sun, Fanfan Li, Bing Zhao, Guohui Han, Zhongsheng Tong, Hua Yang, Yongmei Yin, Xiangshun Kong, Ying Wang, Jianyun Nie, Lixia Ma, Yongqiang Zhang, Jing Luo, Changping Shan, Jing Yao, Shisheng Tan, Xiaoling Ling, Hongmei Sun, Huihui Li, Li Ma, Tao Zhou, Yunjiang Liu, Yixin Qi, Zhenchuan Song, Yuntao Li, Chao Yang, Tianli Hui, Meiqi Wang, Haoqi Wang, Xi Zhang, Wenhui Zhao, Yanhui Li, Mengmeng Li, Ying Xin, Xuan Luo, Deliang Yin, Hongmei Luo, Huan Liu, Qianyun Lu, Jing Yuan, Qingyuan Zhang, Cuizhi Geng

**Affiliations:** ^1^ Breast Center, The Fourth Hospital of Hebei Medical University, Shijiazhuang, Hebei, P. R. China.; ^2^ Department of Medical Oncology, Harbin Medical University Cancer Hospital, Harbin, Heilongjiang, P. R. China.; ^3^ Department of Medical Oncology, Liaoning Cancer Hospital & Institute, Shenyang, Liaoning, P. R. China.; ^4^ Department of Oncology, The Second Hospital of Anhui Medical University, Hefei, Anhui, P. R. China.; ^5^ Department of Medical Oncology, Xinjiang Medical University Affiliated Cancer Hospital, Urumqi, Xinjiang, P. R. China.; ^6^ Department of Medical Oncology, Shanxi Cancer Hospital, Taiyuan, Shanxi, P. R. China.; ^7^ Department of Medical Oncology, Tianjin Medical University Cancer Institute & Hospital, Tianjin, P. R. China.; ^8^ Department of Medical Oncology, Affiliated Hospital of Hebei University, Baoding, Hebei, P. R. China.; ^9^ Department of Oncology, Jiangsu Province Hospital, Nanjing, Jiangsu, P. R. China.; ^10^ Department of Breast Surgery, Xingtai People’s Hospital, Xingtai, Hebei, P. R. China.; ^11^ Department of Breast Surgery, Sun Yat-sen Memorial Hospital, Sun Yat-sen University, Guangzhou, Guangdong, P. R. China.; ^12^ Department of Breast Surgery, Yunnan Cancer Hospital, Kunming, Yunnan, P. R. China.; ^13^ Department of Medical Oncology, Jilin Cancer Hospital, Changchun, Jilin, P. R. China.; ^14^ Department of Medical Oncology, Beijing Hospital, Beijing, P. R. China.; ^15^ Department of Breast Surgery, Sichuan Provincial People’s Hospital, Chengdu, Sichuan, P. R. China.; ^16^ Department of Medical Oncology, Affiliated Hospital of Jining Medical University, Jining, Shandong, P. R. China.; ^17^ Cancer Center, Union Hospital, Tongji Medical College, Huazhong University of Science and Technology, Wuhan, Hubei, P. R. China.; ^18^ Department of Oncology, Guizhou Provincial People’s Hospital, Guiyang, Guizhou, P. R. China.; ^19^ Department of Medical Oncology, The First Hospital of Lanzhou University, Lanzhou, Gansu, P. R. China.; ^20^ Department of Oncology, Jiamusi Tumor Tuberculosis Hospital, Jiamusi, Heilongjiang, P. R. China.; ^21^ Department of Breast Medical Oncology, Shandong Cancer Hospital and Institute, Shandong First Medical University and Shandong Academy of Medical Sciences, Jinan, Shandong, P. R. China.; ^22^ Clinical Development Division, CSPC Ouyi Pharmaceutical Technology Co., Ltd., Shijiazhuang, Hebei, P. R. China.

## Abstract

**Background:** SYHX2011 is a novel albumin-bound paclitaxel, in which most nonparticulate human albumin is replaced with mannitol and sucrose. This study aimed to compare SYHX2011 and paclitaxel for injection (albumin-bound) (PAB) in patients with breast cancer. **Methods:** Patients with histologically or cytologically confirmed unresectable locally advanced or metastatic breast cancer were randomly assigned to receive SYHX2011 (260 mg/m^2^) or PAB (260 mg/m^2^) intravenously once every 3 weeks, stratified by prior taxane use and rash history (prior taxane with rash, prior taxane without rash, or no prior taxane), as well prior lines of chemotherapy for advanced disease (0 or ≥1). The primary endpoint was objective response rate assessed by an independent review committee. Noninferiority was to be declared if the lower bound of the 95% confidence interval (CI) for the rate ratio exceeded 0.75; if the lower bound exceeded 1, superiority would subsequently be tested and considered confirmed. **Results:** In this multicenter, randomized, double-blind, phase III trial across 56 centers in China, 621 patients were screened between 2023 April 23 and 2024 March 21, of whom 459 patients were randomized to SYHX2011 (*n* = 229) or PAB (*n* = 230). The confirmed objective response rate assessed by an independent review committee was 35.8% (95% CI 29.4% to 42.6%) for SYHX2011 and 25.8% (95% CI 20.2% to 32.1%) for PAB (rate ratio = 1.38, 95% CI 1.04 to 1.84; one-sided *P* = 0.012), indicating that SYHX2011 was noninferior to PAB. The superiority of SYHX2011 over PAB was also confirmed. SYHX2011 showed a lower incidence of rash compared with PAB during the first 2 administration cycles (13.6% vs. 34.3%) and all treatment cycles (16.2% vs. 42.6%). Treatment-related adverse events (TRAEs) occurred in 98.2% of patients receiving SYHX2011 and 98.3% of patients receiving PAB. In the SYHX2011 group, 111 (48.7%) patients experienced grade ≥3 TRAEs, compared with 101 (43.9%) patients in the PAB group. The most common grade ≥3 TRAEs were neutropenia, leukopenia, and peripheral sensory neuropathy. The median investigational drug reconstitution time was 2.0 min for SYHX2011 and 11.0 min for PAB. **Conclusions:** SYHX2011 demonstrated greater therapeutic benefits than PAB and significantly reduced the incidence of rash. Additionally, it could offer greater convenience in clinical application, providing advanced breast cancer patients with more effective and safer treatment options. **Trial registration:** ClinicalTrials.gov identifier: NCT05753865. Date of registration: 2023 February 22.

## Background

Female breast cancer is the second most commonly diagnosed cancer and the fourth leading cause of cancer-related mortality worldwide [1]. According to the Global Cancer Observatory (GLOBOCAN) 2022 estimates, about 357,200 new cases and 75,000 deaths from breast cancer occurred in China [2,3]. Approximately 5% to 10% of patients with breast cancer present with metastatic disease at diagnosis [4], and up to 30% of those initially diagnosed with early-stage, nonmetastatic breast cancer will eventually develop distant metastasis [5]. The 5-year survival rate for advanced breast cancer is around 33% [6].

In recent years, despite substantial progress in the development of novel targeted therapies for advanced breast cancer, chemotherapy continues to serve as a cornerstone of treatment. Paclitaxel is a highly effective chemotherapeutic agent and is widely used in the standard management of both early [7] and advanced breast cancer [8]. However, the clinical utility of conventional paclitaxel is limited by its formulation, which relies on Cremophor EL as a solubilizer because of the drug’s poor water solubility. Cremophor EL is associated with increased risk of hypersensitivity reactions [9]. Therefore, routine premedication with corticosteroids is mandatory prior to paclitaxel administration in clinical practice.

Albumin-bound paclitaxel is a Cremophor EL-free formulation of paclitaxel that largely obviates the need for corticosteroid premedication and is associated with a minimal risk of hypersensitivity reactions. An additional proposed advantage of albumin-bound paclitaxel is the potential for enhanced tumor accumulation of paclitaxel via binding to secreted protein acidic and rich in cysteine (SPARC), which exhibits high affinity for albumin and is overexpressed in various tumors [10,11]. Abraxane was the first albumin-bound paclitaxel to receive regulatory approval. Paclitaxel for injection (albumin-bound) (Keaili; PAB), a generic version of Abraxane, was subsequently approved for the treatment of advanced breast cancer in China [12]. However, conventional albumin-bound paclitaxel was associated with a high incidence of skin rash [13] and a prolonged preparation time, highlighting the need for further optimization.

SYHX2011, developed by CSPC Ouyi Pharmaceutical Co., Ltd., is a novel albumin-bound paclitaxel formulation in which the majority of nonparticulate human serum albumin (HSA) is replaced with mannitol and sucrose. This compositional modification is intended to facilitate drug preparation and reduce the incidence of HSA-associated rash [14]. Pharmacokinetic equivalence between SYHX2011 and Abraxane was previously established in patients with advanced breast cancer (NCT05274893), based on comparable exposure levels of total (*C*_max_: 12,414.2 ± 2,175.1 ng/ml vs. 11,646.8 ± 2,224.6 ng/ml; AUC_0–t_ [area under the curve]: 12,833.9 ± 2,847.2 ng·h/ml vs. 13,166.3 ± 3,037.2 ng·h/ml; AUC_0–∞_: 13,191.2 ± 2,856.1 ng·h/ml vs. 13,550.1 ± 3,060.1 ng·h/ml) and free paclitaxel (*C*_max_: 401.6 ± 146.7 ng/ml vs. 349.6 ± 108.5 ng/ml; AUC_0–t_: 209.3 ± 84.5 ng·h/ml vs. 196.0 ± 69.6 ng·h/ml; AUC_0–∞_: 242.3 ± 87.5 ng·h/ml vs. 227.3 ± 67.6 ng·h/ml). The 90% confidence intervals (CIs) for the geometric mean ratios of *C*_max_, AUC_0–t_, and AUC_0–∞_ all fell within the prespecified equivalence margin of 80.00% to 125.00%. The present study aimed to evaluate the efficacy and safety of SYHX2011 compared with those of PAB in patients with advanced breast cancer.

## Materials and Methods

### Study design and participants

This was a multicenter, randomized, double-blind, phase III trial conducted at 56 centers in China (Table S1). Patients with histologically or cytologically confirmed unresectable, locally advanced, or metastatic breast cancer were eligible. Patients were included if they were candidates for single-agent albumin-bound paclitaxel therapy according to Chinese Society of Clinical Oncology Breast Cancer Guidelines 2022 [15] at the investigator’s discretion. Other inclusion criteria included having at least one measurable lesion according to Response Evaluation Criteria in Solid Tumors (RECIST) version 1.1, age 18 years or older, an Eastern Cooperative Oncology Group performance status of 0 to 1, and a life expectancy of at least 3 months. Patients with a history of hypersensitivity reactions to taxanes or HSA (National Cancer Institute Common Terminology Criteria for Adverse Events [NCI-CTCAE] version 5.0, grade ≥3) were excluded. Other key exclusion criteria included any malignancy within 5 years (except carcinoma in situ or basal cell carcinoma), active brain metastasis, or unresolved toxicity from prior antitumor therapy (NCI-CTCAE version 5.0, grade >1). The full inclusion and exclusion criteria are detailed in the Supplementary Materials. This study is registered with ClinicalTrials.gov: NCT05753865.

### Randomization and masking

Patients were randomized in a 1:1 ratio to receive SYHX2011 or PAB using interactive web response systems. Randomization was stratified by prior taxane exposure and history of rash (prior taxanes with rash, prior taxanes without rash, or no prior taxanes), as well as prior lines of chemotherapy for advanced disease (0 or ≥1). Prior taxane exposure was categorized into 2 types based on the treatment context: (a) taxanes administered in the neoadjuvant or adjuvant setting for early-stage breast cancer and (b) taxanes used as first-line treatment for advanced breast cancer. SYHX2011 and PAB were identical in appearance but differed slightly in their solvent. Therefore, while the independent nurses responsible for study drug reconstitution were unblinded due to this difference, all other study staff and patients remained unaware of treatment assignment throughout the study.

### Intervention

SYHX2011 and PAB were administered intravenously over 30 min at a dose of 260 mg/m^2^ on day 1 of each 21-day cycle. All administrations were performed under the supervision of a physician experienced in the use of cancer chemotherapeutic agents. Treatment continued until disease progression, intolerable toxicities, initiation of a new anticancer therapy, withdrawal of consent, loss to follow-up, or death, whichever occurred first. For prophylaxis of vomiting and myelosuppression, patients received an intravenous infusion of ondansetron (8 mg) prior to administration of the study drugs, followed by a subcutaneous infusion of pegylated recombinant human granulocyte colony-stimulating factor (6 mg) 48 h after administration.

A maximum of 2 dose reductions was permitted. Dose interruption was applied in the event of grade 3 neurotoxicity, and treatment was resumed at a reduced dose once the toxicity had resolved to grade ≤2. Dose delay exceeding one treatment cycle was not permitted. The starting dose of SYHX2011 and PAB was 260 mg/m^2^. Dose reduction to 220 mg/m^2^ was required upon occurrence of the following adverse events (AEs): severe neutropenia (absolute neutrophil count <0.5 × 10^9^/l for ≥1 week) or severe peripheral sensory neuropathy. If these AEs recurred despite the first dose reduction, the dose was further reduced from 220 to 180 mg/m^2^.

### Outcomes and assessment

The primary endpoint was confirmed objective response rate (ORR) as assessed by an independent review committee (IRC) according to RECIST version 1.1. The key secondary endpoint was the incidence of rash during the first 2 administration cycles as well as all treatment cycles. Other secondary endpoints included investigator-assessed confirmed ORR, progression-free survival (PFS), time to progression (TTP), overall survival (OS), safety, and reconstitution time (time to complete dissolution completeness) of the study drug.

Tumor assessments were performed according to RECIST version 1.1 using enhanced computed tomography or magnetic resonance imaging every 6 weeks until disease progression, withdrawal of consent, loss to follow-up, death, or initiation of a new anticancer therapy, whichever occurred first. Survival follow-up was conducted every 12 weeks after the end of treatment. Tumor response, based on IRC-assessed best overall response, included complete response (CR), partial response (PR), stable disease (SD), progressive disease, and not evaluable. ORR was defined as the proportion of patients with a best overall response of CR or PR. Disease control rate (DCR) was defined as the proportion of patients with a confirmed best overall response of CR, PR, or SD.

Safety assessment included monitoring of AEs, vital signs, physical examination, laboratory parameters, electrocardiograms, and left ventricular ejection fraction. These assessments continued for 28 d after the last dose or until the initiation of a new anticancer therapy, whichever occurred first. AEs were coded using Medical Dictionary for Regulatory Activities version 27.0 and graded according to NCI-CTCAE version 5.0.

### Statistical analysis

The primary endpoint of IRC-assessed ORR was tested for noninferiority first, and if noninferiority was established, superiority was subsequently tested. Assuming an ORR of 49% in the PAB group based on 2 previous studies [13,16], the rate ratio (RR; SYHX2011 vs. PAB) was calculated. Noninferiority would be declared if the lower bound of the 95% CI for RR exceeded the prespecified noninferiority margin of 0.75. Superiority would be concluded if the lower bound of the 95% CI exceeded 1. A total of 410 patients were expected to provide 81.3% power at a one-sided alpha level of 0.025 using a noninferiority margin of 0.75. Therefore, considering that approximately 10% of the enrolled patients would be unavailable for tumor assessments, the target number of patients was set at 445.

Efficacy was assessed in the intention-to-treat (ITT) population, comprising all randomized participants, and the modified ITT (mITT) population, defined as all randomized participants who received at least one dose of the study drug and had both baseline and at least one postbaseline efficacy assessment. Safety was evaluated in the safety analysis set, which included all patients who received at least one dose of the study drug. The Clopper–Pearson method was used to calculate ORR and DCR with corresponding 95% CIs for both groups. The RRs for ORR between the 2 groups and its 95% CI were examined using the Cochran–Mantel–Haenszel method. PFS, TTP, and OS were analyzed using the Kaplan–Meier method, with median times and corresponding 95% CIs, as well as 3-, 6-, 9-, and 12-month rates with 95% CI. The hazard ratio (HR) with 95% CI was determined using the Cox proportional hazards model. Prespecified subgroup analysis of the best overall response rate was planned according to molecule subtype (hormone receptor [HR] positive or triple-negative breast cancer [TNBC]), prior lines of chemotherapy for advanced disease (0 or ≥1), prior taxane exposure (yes or no), and presence of liver metastasis (yes or no). The incidence of rash in each group was calculated using the Clopper–Pearson method with 95% CI, and the RR for rash incidence between the 2 groups was examined using the Cochran–Mantel–Haenszel method. A post hoc analysis evaluated the efficacy of SYHX2011 versus that of PAB according to line of therapy, stratified by patients receiving study drugs as first-line therapy and those receiving study drugs as second- or later-line therapy. All statistical analyses were performed using SAS software version 9.4.

## Results

### Patients and disease characteristics

**Fig. 1.**
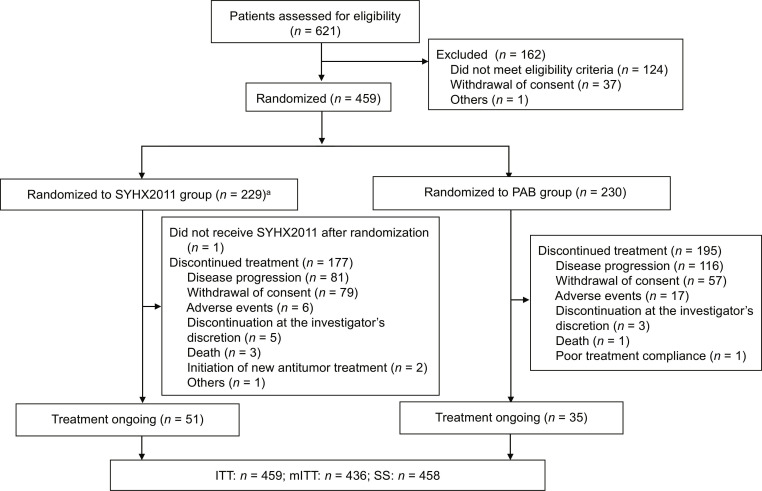
Study profile. At the cutoff date of 2024 September 21, 459 patients were enrolled. ^a^One patient did not receive SYHX2011 after randomization. ITT, intention to treat; mITT, modified intention to treat; SS, safety analysis set.

Between 2023 April 23 and 2024 March 21, 621 patients were screened for eligibility, of whom 459 patients were enrolled and randomly assigned to receive SYHX2011 (*n* = 229) or PAB (*n* = 230). The main reasons for screening failure were not meeting eligibility criteria (*n* = 124) and withdrawal of consent (*n* = 37) (Fig. 1).

Baseline demographic and disease characteristics were well balanced between the 2 treatment groups (Table 1). In both the SYHX2011 and PAB groups, the predominant molecular subtype was HR-positive breast cancer (75.5% vs. 72.2%), followed by TNBC (24.5% vs. 27.8%). The majority of patients had received prior taxane therapy (70.3% vs. 69.5%); only 3 patients (1.3%) in the SYHX2011 group and 1 patient (0.4%) in the PAB group had a history of rash following prior taxane treatment. In the SYHX2011 and PAB groups, 165 (72.1%) and 167 (72.6%) patients received the study drugs as first-line chemotherapy, while 64 (27.9%) and 63 (27.4%) received them as second- or later-line chemotherapy, respectively. Additional baseline characteristics were comparable between patients in the 2 groups (Tables S2 and S3). As of the data cutoff date (2024 September 21), the median follow-up duration was 8.8 (95% CI 8.3 to 9.6) months in the SYHX2011 group and 9.1 (95% CI 8.4 to 9.6) months in the PAB group.

**Table 1.** Demographic and clinical characteristics at baseline

Characteristics	SYHX2011 (*n* = 229)	PAB (*n* = 230)
Age, years		
Median (range)	57.0 (27.0–84.0)	56.0 (28.0–84.0)
<65, *n* (%)	183 (79.9)	187 (81.3)
≥65, *n* (%)	46 (20.1)	43 (18.7)
Sex, *n* (%)		
Male	2 (0.9)	0 (0.0)
Female	227 (99.1)	230 (100)
ECOG PS, *n* (%)		
0	85 (37.1)	95 (41.3)
1	144 (62.9)	135 (58.7)
Menopause ^a^, *n* (%)		
Yes	177 (77.3)	172 (74.8)
No	50 (21.8)	58 (25.2)
Breast cancer molecular subtype, *n* (%)		
HR-positive ^b^ BC	173 (75.5)	166 (72.2)
TNBC	56 (24.5)	64 (27.8)
Clinical stage, *n* (%)		
Stage IV at diagnosis ^c^	138 (60.3)	126 (54.8)
Relapsed or metastatic	46 (20.1)	61 (26.5)
Unknown and others	45 (19.6)	43 (18.7)
Metastatic organs ^d^, *n* (%)		
Bone	136 (59.4)	140 (60.9)
Lymph nodes	117 (51.1)	109 (47.4)
Lung	108 (47.2)	102 (44.3)
Liver	105 (45.9)	109 (47.4)
Brain	3 (1.3)	7 (3.0)
Others	74 (32.3)	75 (32.6)
Prior lines of chemotherapy for advanced disease, *n* (%)		
0	165 (72.1)	167 (72.6)
≥1	64 (27.9)	63 (27.4)
Prior taxane exposure ^e^ and history of rash, *n* (%)		
Prior taxanes with rash	3 (1.3)	1 (0.4)
Prior taxanes without rash	158 (69.0)	159 (69.1)
No prior taxanes	68 (29.7)	70 (30.4)
Prior treatment with CDK4/6 inhibitor, *n* (%)		
Yes	75 (32.8)	58 (25.2)
No	137 (59.8)	136 (59.1)
Unknown	17 (7.4)	36 (15.7)

BC, breast cancer; ECOG PS, Eastern Cooperative Oncology Group performance status; HR, hormone receptor; PAB, paclitaxel for injection (albumin-bound); TNBC, triple-negative breast cancer

^a^ 2 patients in the SYHX2011 group were male.

^b^ HR-positive BC was defined as tumors with >1% of cells staining positive for estrogen receptors and/or progesterone receptors by immunohistochemistry.

^c^ TNM staging system.

^d^ As patients can present with metastases in more than one organ, the summed percentages exceed the total number of patients with organ involvement.

^e^ 246 patients (76.6%) had prior taxane exposure in the neoadjuvant or adjuvant setting, and 73 patients (22.7%) had received taxanes in the advanced disease setting.

### Treatment exposure

The median exposure to study drugs was 7.1 (interquartile range 4.0 to 10.6) cycles for SYHX2011 and 6.4 (interquartile range 3.1 to 10.1) cycles for PAB. The mean ± standard deviation relative dose intensity was 98.3% ± 20.3% for SYHX2011 and 95.9% ± 7.5% for PAB. Dose reduction occurred in 27 (11.8%) patients in the SYHX2011 group and 36 (15.7%) patients in the PAB group, while dose interruptions occurred in 63 (27.6%) patients and 66 (28.7%) patients, respectively (Table S4). At the data cutoff date, 372 patients discontinued treatment, with disease progression being the most frequently reported reason (*n* = 197) (Fig. 1).

### Efficacy

In the mITT population, the confirmed IRC-assessed ORR was 35.8% (77/215; 95% CI 29.4% to 42.6%) for SYHX2011 and 25.8% (57/221; 95% CI 20.2% to 32.1%) for PAB (Table 2). Noninferiority of SYHX2011 versus PAB was met (RR = 1.38, 95% CI 1.04 to 1.84; *P* = 0.012), as the lower bound of 95% CI exceeded the predefined noninferiority margin of 0.75. Superiority was subsequently established, as the lower bound of the 95% CI also exceeded 1.00. The trends in ORR outcomes observed with SYHX2011 compared to PAB in both patients who received study drugs as first-line chemotherapy and patients who received study drugs as second- or later-line chemotherapy were consistent with or similar to those in the overall population. Results from the ITT analysis aligned with those of the mITT analysis, reinforcing the robustness of the primary endpoint (Table 2 and Tables S5 and S6). The confirmed ORRs assessed by the investigator in both mITT and ITT populations were also consistent with the IRC-assessed ORR (Table 2 and Tables S5 and S6). The improvement in confirmed ORR with SYHX2011 compared to PAB was consistently observed across all predefined subgroups, including the mITT population, mITT patients who received study drugs as first-line chemotherapy, and mITT patients who received study drugs as second- or later-line chemotherapy (Figs. S1 to S3).

**Table 2.** Summary of efficacy assessed by the IRC and investigator in the mITT and ITT populations

Best overall response, *n* (%)	mITT				ITT			
	IRC assessed		Investigator assessed		IRC assessed		Investigator assessed	
	SYHX2011 (*n* = 215)	PAB (*n* = 221)	SYHX2011 (*n* = 215)	PAB (*n* = 221)	SYHX2011 (*n* = 229)	PAB (*n* = 230)	SYHX2011 (*n* = 229)	PAB (*n* = 230)
CR	0 (0.0)	2 (0.9)	3 (1.4)	3 (1.4)	0 (0.0)	2 (0.9)	3 (1.3)	3 (1.3)
PR	77 (35.8)	55 (24.9)	78 (36.3)	59 (26.7)	77 (33.6)	55 (23.9)	78 (34.1)	59 (25.7)
SD	106 (49.3)	122 (55.2)	104 (48.4)	120 (54.3)	106 (46.3)	122 (53.0)	104 (45.4)	120 (52.2)
PD	28 (13.0)	37 (16.7)	28 (13.0)	34 (15.4)	28 (12.2)	37 (16.1)	28 (12.2)	34 (14.8)
NE	4 (1.9)	5 (2.3)	2 (0.9)	5 (2.3)	18 (7.9)	14 (6.1)	16 (7.0)	14 (6.1)
ORR (95% CI), %	35.8 (29.4–42.6)	25.8 (20.2–32.1)	37.7 (31.2–44.5)	28.1 (22.2–34.5)	33.6 (27.5–40.1)	24.8 (19.3–30.9)	35.4 (29.2–41.9)	27.0 (21.3–33.2)
DCR (95% CI), %	85.1 (79.6–89.6)	81.0 (75.2–86.0)	86.0 (80.7–90.4)	82.4 (76.7–87.1)	79.9 (74.1–84.9)	77.8 (71.9–83.0)	80.8 (75.1–85.7)	79.1 (73.3–84.2)

CI, confidence interval; CR, complete response; DCR, disease control rate; IRC, independent review committee; ITT, intention to treat; mITT, modified intention to treat; NE, not evaluable; ORR, objective response rate; PAB, paclitaxel for injection (albumin-bound); PD, progressive disease; PR, partial response; SD, stable disease

**Fig. 2.**
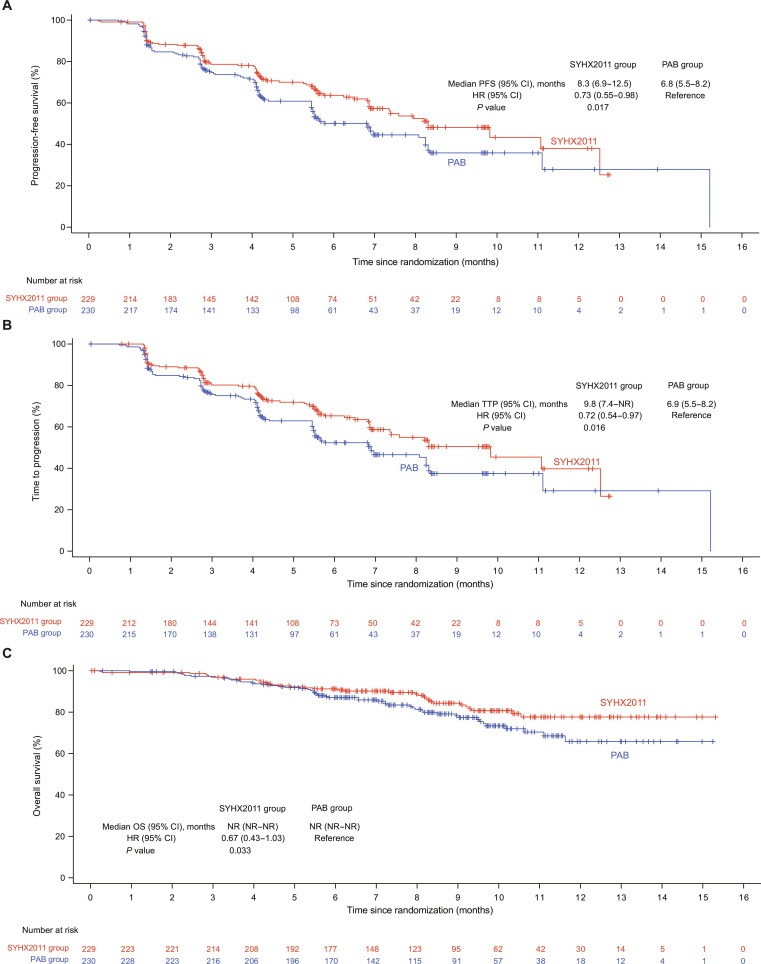
Kaplan–Meier estimates of survival in the ITT population. (A) Progression-free survival. (B) Time to progression. (C) Overall survival. CI, confidence interval; HR, hazard ratio; ITT, intention to treat; NR, not reached; OS, overall survival; PFS, progression-free survival; TTP, time to progression.

Median PFS was longer with SYHX2011 than with PAB in the ITT population (8.3 months vs. 6.8 months, Fig. 2A), corresponding to a 27% reduction in the risk of disease progression or death with SYHX2011 compared to PAB (HR = 0.73, 95% CI 0.55 to 0.98; *P* = 0.017). A similar trend was seen in ITT patients receiving study drugs as first-line chemotherapy (9.8 months vs. 6.9 months; Fig. S4) and in ITT patients receiving them as second- or later-line chemotherapy (7.4 months vs. 5.5 months; Fig. S5). The median TTP was 9.8 months in the SYHX2011 group compared to 6.9 months in the PAB group in the ITT population (HR = 0.72, 95% CI 0.54 to 0.97; *P* = 0.016; Fig. 2B). Similar trends were seen in ITT patients receiving study drugs as first-line chemotherapy (9.8 months vs. 7.0 months; Fig. S6) and in ITT patients receiving study drugs as second- or later-line chemotherapy (8.2 months vs. 5.5 months; Fig. S7). At the time of data cutoff, OS was not mature, with median OS not reached in either group; therefore, OS results are exploratory and no definitive survival benefit can be concluded. SYHX2011 showed a trend toward improved OS compared with PAB in the ITT population (HR = 0.67, 95% CI 0.43 to 1.03; *P* = 0.033), as well as in ITT patients receiving study drugs as first-line chemotherapy and those who received study drugs as second- or later-line chemotherapy (Fig. 2C and Figs. S8 and S9).

### Safety

**Fig. 3.**
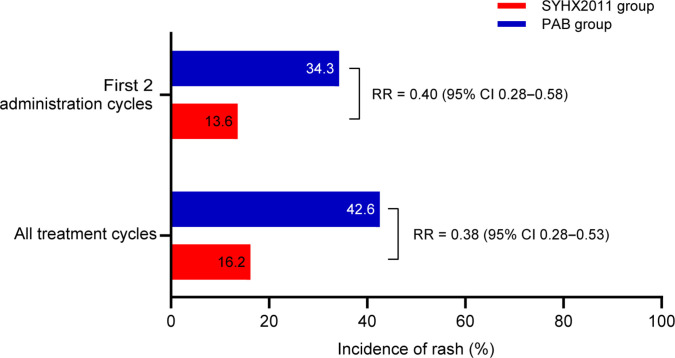
Incidence of rash in the SYHX2011 and PAB groups during the first 2 administration cycles and all treatment cycles. CI, confidence interval; PAB, paclitaxel for injection (albumin-bound); RR, rate ratio.

SYHX2011 was associated with a lower incidence of rash during the first 2 treatment cycles compared with PAB (13.6% vs. 34.3%), with an adjusted RR of 0.40 (95% CI 0.28 to 0.58; one-sided *P* < 0.001; Fig. 3). A similar reduction in rash incidence was also observed during all treatment cycles (16.2% vs. 42.6%; adjusted RR = 0.38, 95% CI 0.28 to 0.53; *P* < 0.001; Fig. 3).

In the safety analysis set, treatment-related adverse events (TRAEs) occurred in 98.2% of patients receiving SYHX2011 and 98.3% of patients receiving PAB. Grade ≥3 TRAEs were reported in 111 (48.7%) patients in the SYHX2011 group and 101 (43.9%) patients in the PAB group (Table 3). The most common (occurring in ≥5% of patients in either group) grade ≥3 TRAEs were neutropenia (15.8% vs. 11.3%), leukopenia (14.0% vs. 11.7%), peripheral sensory neuropathy (10.5% vs. 10.4%), lymphopenia (9.2% vs. 10.0%), anemia (6.1% vs. 6.5%), and thrombocytopenia (5.3% vs. 4.3%). Serious treatment-emergent adverse events (TEAEs) occurred in 28 of 228 patients (12.3%) receiving SYHX2011 and 40 of 230 patients (17.4%) receiving PAB (Table S7).

**Table 3.** Summary of adverse events in the patients treated with SYHX2011 or PAB

Incidence, *n* (%)	SYHX2011 (*n* = 228)		PAB (*n* = 230)	
	All grades	Grade ≥3	All grades	Grade ≥3
TEAEs	225 (98.7)	115 (50.4)	227 (98.7)	111 (48.3)
TRAEs	224 (98.2)	111 (48.7)	226 (98.3)	101 (43.9)
Common TRAEs ^a^				
Anemia	131 (57.5)	14 (6.1)	138 (60.0)	15 (6.5)
Leukopenia	109 (47.8)	32 (14.0)	93 (40.4)	27 (11.7)
Thrombocytopenia	100 (43.9)	12 (5.3)	88 (38.3)	10 (4.3)
Peripheral sensory neuropathy	93 (40.8)	24 (10.5)	90 (39.1)	24 (10.4)
Alopecia	87 (38.2)	0 (0.0)	99 (43.0)	0 (0.0)
Increased ALT	85 (37.3)	3 (1.3)	89 (38.7)	3 (1.3)
Neutropenia	85 (37.3)	36 (15.8)	72 (31.3)	26 (11.3)
Increased AST	82 (36.0)	5 (2.2)	85 (37.0)	3 (1.3)
Pruritus	59 (25.9)	1 (0.4)	54 (23.5)	0 (0.0)
Fatigue	54 (23.7)	4 (1.8)	62 (27.0)	4 (1.7)
γ-GGT elevation	47 (20.6)	5 (2.2)	41 (17.8)	2 (0.9)
Nausea	47 (20.6)	0 (0.0)	40 (17.4)	0 (0.0)
Lymphopenia	44 (19.3)	21 (9.2)	47 (20.4)	23 (10.0)
Weight loss	42 (18.4)	0 (0.0)	55 (23.9)	1 (0.4)
Hyperuricemia	41 (18.0)	0 (0.0)	37 (16.1)	1 (0.4)
Peripheral neuropathy	35 (15.4)	6 (2.6)	32 (13.9)	4 (1.7)
Increased ALP	35 (15.4)	0 (0.0)	41 (17.8)	0 (0.0)
Vomiting	31 (13.6)	2 (0.9)	35 (15.2)	0 (0.0)
Pruritic rash	29 (12.7)	1 (0.4)	86 (37.4)	9 (3.9)

ALP, alkaline phosphatase; ALT, alanine aminotransferase; AST, aspartate aminotransferase; GGT, glutamyl transferase; PAB, paclitaxel for injection (albumin-bound); TEAEs, treatment-emergent adverse events; TRAEs, treatment-related adverse events

^a^ Common TRAEs are defined as TRAEs that occurred in at least 10% of the patients.

TEAEs leading to permanent treatment discontinuation were reported in 5 patients (2.2%) receiving SYHX2011 and 16 patients (7.0%) receiving PAB (Table S8). The most common TEAEs leading to discontinuation (occurring in ≥1% of patients in either group) were peripheral sensory neuropathy in the SYHX2011 group (3 cases) and peripheral sensory neuropathy (4 cases) and peripheral neuropathy (3 cases) in the PAB group. TEAEs leading to dose reduction or dose interruption occurred in 28 (12.3%) and 54 (23.7%) patients receiving SYHX2011 and 37 (16.1%) and 56 (24.3%) patients receiving PAB.

In the SYHX2011 group, 4 (1.8%) patients experienced TEAEs leading to death, of whom 3 were considered treatment related (one case each of infectious pneumonia, infectious shock, and sepsis) (Table S8). The fourth patient died of an unknown cause at home. In the PAB group, 3 (1.3%) patients experienced TEAEs leading to death (one case each of infectious pneumonia, dyspnea, and unknown cause); none of these events were deemed treatment related. No new safety signals were observed in the SYHX2011 group.

### Drug reconstitution

The median investigational drug reconstitution time was 2.0 (range 0.1 to 28.2) min for SYHX2011 and 11.0 (range 1.1 to 38.8) min for PAB. In the SYHX2011 group, 50.0% (114/228) of patients had a preparation time of ≤2 min, compared with 0.4% (1/230) in the PAB group.

## Discussion

In this study, SYHX2011 met the primary endpoint of noninferiority in ORR and demonstrated superior ORR compared with PAB in patients with advanced breast cancer. SYHX2011 also showed statistically significant improvements in PFS and TTP compared with PAB as assessed by both the IRC and investigators. Furthermore, SYHX2011 showed a favorable trend toward improved OS compared with PAB. In addition, SYHX2011 was associated with a lower incidence of rash and a shorter reconstitution time than PAB.

Chemotherapy remains a cornerstone in the treatment of breast cancer across all stages, both as monotherapy [17,18] and in combination with other therapies [19,20]. In a phase III clinical trial involving patients with TNBC, first-line treatment with albumin-bound paclitaxel plus platinum significantly improved median PFS and OS compared with gemcitabine plus platinum [21]. Moreover, multiple studies have demonstrated that combining immune checkpoint inhibitors with albumin-bound paclitaxel improves median PFS and OS compared with albumin-bound paclitaxel monotherapy in patients with programmed death-ligand 1 (PD-L1)-positive TNBC [20,22,23]. In the neoadjuvant setting for human epidermal growth factor receptor 2 (HER2)-positive early breast cancer, a recent study showed that albumin-bound paclitaxel plus trastuzumab and pertuzumab resulted in a higher pathological complete response rate compared with docetaxel and carboplatin plus trastuzumab and pertuzumab [24]. Additionally, an anthracycline- and carboplatin-free neoadjuvant regimen featuring albumin-bound paclitaxel, trastuzumab, and pertuzumab demonstrated comparable efficacy with reduced treatment-related toxicities in patients with HER2-positive breast cancer [25]. These findings suggest a promising role for SYHX2011 in future therapeutic strategies for advanced breast cancer.

Previous studies have established the efficacy of the original albumin-bound paclitaxel formulation (Abraxane) in patients with metastatic breast cancer. A phase II study of Abraxane enrolling 63 patients with metastatic breast cancer reported an ORR of 48%, with a median TTP of 6.1 months and a median OS of 14.6 months [26]. In the CA012 study, Abraxane demonstrated superior efficacy compared with standard paclitaxel, with ORR increasing from 19% to 33% (*P* = 0.001) and median TTP increasing from 3.9 to 5.3 months (HR 0.75, *P* = 0.006) [27]. The ORR with Abraxane was comparable to that with SYHX2011 in the overall patient population (33.2% vs. 35.8%) and in those who received study drugs as first-line chemotherapy (42.3% vs. 39.2%) [27]. Although cross-trial comparisons should be interpreted with caution, median TTP appeared numerically longer with SYHX2011 than with Abraxane among patients receiving study drugs as first-line chemotherapy (9.8 months vs. 5.5 months) and those receiving study drugs as second- or later-line chemotherapy (8.2 months vs. 4.8 months). The clinical utility of Abraxane is complicated by its prolonged reconstitution time and propensity to foam, necessitating special handling procedures. These issues stem from the formulation’s properties: the lyophilized albumin shields hydrophilic groups, delaying reconstitution, and its inherent amphiphilicity acts as a surfactant, promoting foam formation during agitation. Consequently, its use in clinical applications is associated with extended preparation time and the need for specialized handling procedures to prevent foaming. SYHX2011 is an innovative formulation in which the excess albumin in Abraxane is removed during manufacturing, and mannitol is added as a lyoprotectant. As a result, SYHX2011 addresses the limitations associated with Abraxane, including complex preparation procedures and prolonged reconstitution time. It also avoids problems like excessive foam and incomplete dissolution that may result from improper handling. Collectively, these improvements enhance ease of preparation and mitigate the risks associated with dissolution-related challenges during administration.

Rash is a more common AE following albumin-bound paclitaxel treatment compared with conventional paclitaxel [13,14,28] and may necessitate dose reduction or interruption. Severe rash can also impose a serious psychological burden, reduce the quality of life, and affect treatment compliance. Asian ethnicity and a history of allergy have been identified as risk factors for developing rash after albumin-bound paclitaxel treatment [29]. Therefore, reducing the incidence of rash might significantly improve patient compliance and therapeutic outcomes. In the present study, rash occurred in 16.2% of patients treated with SYHX2011, a rate lower than that observed with Abraxane (27.0%) [13]. Prior data have shown that the incidence of rash associated with albumin-bound paclitaxel is significantly higher in Chinese patients than in Western populations, whereas no such difference is observed with conventional solvent-based paclitaxel [30]. Based on this, the albumin component itself was proposed as a potential contributor to this cutaneous adverse reaction [30]. Although this remains a hypothesis, the reduced rash incidence observed with SYHX2011, which contains a lower HSA content, lends indirect support to this proposition. However, as this study was not designed to investigate mechanisms, these hypotheses warrant validation in future translational research. Nevertheless, our findings suggested that compared with other albumin-bound paclitaxel formulations, SYHX2011 could offer greater efficacy and improved quality of life in patients with advanced breast cancer.

The typical adverse effects associated with albumin-bound paclitaxel included hematologic toxicities such as leukopenia, neutropenia, and anemia, as well as nonhematologic toxicities including peripheral neuropathy, diarrhea, nausea, vomiting, fatigue, and alopecia. In the present study, the most common TRAEs were anemia (57.5%), leukopenia (47.8%), thrombocytopenia (43.9%), peripheral sensory neuropathy (40.8%), alopecia (38.2%), increased alanine aminotransferase (37.3%), neutropenia (37.3%), and increased aspartate aminotransferase (36.0%). In the CA012 study, the most frequent AEs in patients with metastatic breast cancer in the Abraxane group included alopecia, sensory neuropathy, fatigue, neutropenia, arthralgia, myalgia, nausea, infection, and diarrhea, indicating a generally similar safety profile. Of note, the incidence of grade ≥3 neutropenia in the SYHX2011 group was 15.8%, comparing favorably with the historical incidence reported for Abraxane (42%) [13]. In addition, peripheral sensory and motor neuropathy are well-recognized toxicities associated with paclitaxel therapy. In the Cancer and Leukemia Group B trial, grade ≥3 sensory neuropathy was reported in 32.9% of patients receiving conventional paclitaxel at 250 mg/m^2^ [31]. In contrast, the incidence was lower with Abraxane (10.5%) in a prior phase III study [27], and a similarly low rate (10.5%) was observed with SYHX2011 in the current study. Furthermore, in this study, peripheral sensory neuropathy led to permanent treatment discontinuation in only 3 patients (1.3%), dose interruption in 9 patients (3.9%), and dose reduction in 16 patients (6.9%) in the SYHX2011 group. Three infection-related deaths occurred in the SYHX2011 group, which may be associated with grade ≥3 neutropenia. Therefore, close monitoring of neutrophil counts and proactive management of neutropenia are recommended during treatment with this agent. Overall, no new safety signals were identified with SYHX2011, and its AE profiles were consistent with those of other albumin-bound paclitaxel.

The shorter reconstitution time of SYHX2011 (2 min vs. 11 min for PAB) may offer several practical benefits in real-world clinical settings. First, it can improve workflow efficiency by reducing the time and effort required from nursing staff for drug preparation in the pharmacy or infusion suite, particularly during peak hours, thereby potentially alleviating workload. Second, in busy oncology infusion units, cumulative time savings from reduced waiting times can translate into improved patient throughput and more efficient utilization of healthcare resources.

This study has several limitations. First, this study exclusively enrolled Chinese participants; whether these findings are generalizable to other ethnic populations requires further validation. Second, a head-to-head comparison with Abraxane was not feasible as Abraxane has been unavailable in China since 2020 due to Good Manufacturing Practice of Medical Products compliance issues. PAB, a generic version of Abraxane, had been approved for the treatment of advanced breast cancer in China for 6 years based on its equivalence to Abraxane [12]. PAB has shown promising efficacy in patients with advanced breast cancer in clinical studies, consistent with our clinical practice; therefore, it was selected as the comparator in this study. Finally, direct patient- or provider-reported outcome data regarding the potential benefits of the shortened reconstitution time were not collected. Future studies are warranted to prospectively evaluate the clinical impact of this operational advantage.

## Conclusion

In summary, SYHX2011 demonstrates superior therapeutic efficacy compared with PAB and is associated with a significantly lower incidence of rash. Additionally, its ease of preparation in clinical practice provides patients with advanced breast cancer a new treatment option that is more effective, safer, and simpler to use.

## Ethical Approval

The study was approved by the Independent Ethics Committee at each study site and conducted in accordance with the Declaration of Helsinki and Good Clinical Practices. Written informed consent was obtained from all patients before enrollment.

## Data Availability

The datasets (including deidentified individual data) generated during the current study are available from the corresponding authors upon request by contacting Cuizhi Geng (46300349@hebmu.edu.cn) and are not for commercial use. All requests will be reviewed by the corresponding authors and the sponsor, CSPC Ouyi Pharmaceutical Technology Co., Ltd. A signed data access agreement with the sponsor is required before data sharing.
