# Supplementary material for: Albumin-Bound Paclitaxel (SYHX2011) in Patients with Advanced Breast Cancer: A Multicenter, Randomized, Double-Blind, Phase III Study
Source: Cancer Commun (Lond). 2026 Jun 26;46:0037. doi: 10.34133/cancomm.0037 (PMC13305025; doi:10.34133/cancomm.0037)
Supplement: Supplementary 1 — Supplementary Text Figs. S1 to S9 Tables S1 to S8 [file cancomm.0037.f1.docx]

**Supplementary Materials for**

# Albumin-Bound Paclitaxel (SYHX2011) in Patients with Advanced Breast Cancer: A Multicenter, Randomized, Double-blind, Phase III Study

**Authors:**

Lina Zhang^1, †^, Jingxuan Wang^2, †^, Tao Sun^3^, Fanfan Li^4^, Bing Zhao^5^, Guohui Han^6^, Zhongsheng Tong^7^, Hua Yang^8^, Yongmei Yin^9^, Xiangshun Kong^10^, Ying Wang^11^, Jianyun Nie^12^, Lixia Ma^13^, Yongqiang Zhang^14^, Jing Luo^15^, Changping Shan^16^, Jing Yao^17^, Shisheng Tan^18^, Xiaoling Ling^19^, Hongmei Sun^20^, Huihui Li^21^, Li Ma^1^, Tao Zhou^1^, Yunjiang Liu^1^, Yixin Qi^1^, Zhenchuan Song^1^, Yuntao Li^1^, Chao Yang^1^, Tianli Hui^1^, Meiqi Wang^1^, Haoqi Wang^1^, Xi Zhang^1^, Wenhui Zhao^2^, Yanhui Li^22^, Mengmeng Li^22^, Ying Xin^22^, Xuan Luo^22^, Deliang Yin^22^, Hongmei Luo^22^, Huan Liu^22^, Qianyun Lu^22^, Jing Yuan^22^, Qingyuan Zhang^2, *^, Cuizhi Geng^1, *^

**Affiliations:**

^1^Breast Center, The Fourth Hospital of Hebei Medical University, Shijiazhuang, Hebei, P. R. China.

^2^Department of Medical Oncology, Harbin Medical University Cancer Hospital, Harbin, Heilongjiang, P. R. China.

^3^Department of Medical Oncology, Liaoning Cancer Hospital & Institute, Shenyang, Liaoning, P. R. China.

^4^Department of Oncology, The Second Hospital of Anhui Medical University, Hefei, Anhui, P. R. China.

^5^Department of Medical Oncology, Xinjiang Medical University Affiliated Cancer Hospital, Urumqi, Xinjiang, P. R. China.

^6^Department of Medical Oncology, Shanxi Cancer Hospital, Taiyuan, Shanxi, P. R. China.

^7^Department of Medical Oncology, Tianjin Medical University Cancer Institute & Hospital, Tianjin, P. R. China.

^8^Department of Medical Oncology, Affiliated Hospital of Hebei University, Baoding, Hebei, P. R. China.

^9^Department of Oncology, Jiangsu Province Hospital, Nanjing, Jiangsu, P. R. China.

^10^Department of Breast Surgery, Xingtai People's Hospital, Xingtai, Hebei, P. R. China.

^11^Department of Breast Surgery, Sun Yat-sen Memorial Hospital, Sun Yat-sen University, Guangzhou, Guangdong, P. R. China.

^12^Department of Breast Surgery, Yunnan Cancer Hospital, Kunming, Yunnan, P. R. China.

^13^Department of Medical Oncology, Jilin Cancer Hospital, Changchun, Jilin, P. R. China.

^14^Department of Medical Oncology, Beijing Hospital, Beijing, P. R. China.

^15^Department of Breast Surgery, Sichuan Provincial People's Hospital, Chengdu, Sichuan, P. R. China.

^16^Department of Medical Oncology, Affiliated Hospital of Jining Medical University, Jining, Shandong, P. R. China.

^17^Cancer Center, Union Hospital, Tongji Medical College, Huazhong University of Science and Technology, Wuhan, Hubei, P. R. China.

^18^Department of Oncology, Guizhou Provincial People's Hospital, Guiyang, Guizhou, P. R. China.

^19^Department of Medical Oncology, The First Hospital of Lanzhou University, Lanzhou, Gansu, P. R. China.

^20^Department of Oncology, Jiamusi Tumor Tuberculosis Hospital, Jiamusi, Heilongjiang, P. R. China.

^21^Department of Breast Medical Oncology, Shandong Cancer Hospital and Institute, Shandong First Medical University and Shandong Academy of Medical Sciences, Jinan, Shandong, P. R. China.

^22^Clinical Development Division, CSPC Ouyi Pharmaceutical Technology Co., Ltd, Shijiazhuang, Hebei, P. R. China.

***Corresponding author:**

1. Cuizhi Geng

Breast Center, The Fourth Hospital of Hebei Medical University,

No. 169 Tianshan Street, Shijiazhuang, 050011, Hebei, P. R. China.

Email: 46300349@hebmu.edu.cn

2. Qingyuan Zhang

Department of Medical Oncology, Harbin Medical University Cancer Hospital,

No. 150 Haping Road, Harbin, 150086, Heilongjiang, P. R. China

Email: 13313612989@163.com

^†^Lina Zhang and Jingxuan Wang contributed equally to this work.

**Supplementary Materials**

**Inclusion criteria**

1. Eligible subjects must voluntarily participate, provide written informed consent, and be willing and able to comply with all protocol-required visits and procedures;
2. Histologically or cytologically confirmed breast cancer meeting the following conditions:
3. Subjects with unresectable locally advanced or metastatic breast cancer deemed suitable for albumin-bound paclitaxel monotherapy by investigators per Chinese Society of Clinical Oncology (CSCO) Breast Cancer Guidelines (2022).
4. At least one measurable lesion per Response Evaluation Criteria in Solid Tumors (RECIST) version 1.1 criteria. Previously irradiated lesions can only be considered measurable if showing definitive progression post-radiotherapy.
5. Aged ≥18 years;
6. Eastern Cooperative Oncology Group (ECOG) performance status 0 or 1 within 7 days prior to the first dose;
7. Expected survival ≥3 months;
8. Normal major organ function within 7 days before first dose [no blood transfusion, erythropoietin (EPO), granulocyte colony-stimulating factor (G-CSF) or other hematopoietic growth factor therapies within 14 days prior], meeting:
9. Hematology tests: Hemoglobin (Hb) ≥90 g/L; absolute neutrophil count (ANC) ≥1.5×10^9^/L; platelet count (PLT) ≥100×10^9^/L;
10. Chemistry: [Aspartate aminotransferase](https://www.mayoclinic.org/tests-procedures/aspartate-aminotransferase-ast-test/about/pac-20583406) (AST) and alanine aminotransferase (ALT) ≤10 × upper limit of normal (ULN); Total bilirubin (TBiL) ≤1.5 × ULN; Serum creatinine ≤1.5 × ULN with Cockcroft-Gault formula-calculated creatinine clearance ≥30 mL/min;
11. Coagulation function: Activated partial thromboplastin time (APTT) ≤1.5 × ULN, prothrombin time (PT) or international normalized ratio (INR) ≤1.5 × ULN (subjects receiving anticoagulant therapy with coagulation parameters within therapeutic ranges and judged by investigators as not requiring new medications or dose adjustments during treatment may also be enrolled);
12. Subjects of childbearing potential must agree to use reliable contraception [e.g., intrauterine device (IUD), oral contraceptives, or condoms] during the study and for 6 months after study completion; females of childbearing potential must have negative serum pregnancy test within 7 days prior to planned enrollment.

**Exclusion Criteria**

1. History of severe allergy or significant hypersensitivity to paclitaxel drugs or human albumin (National Cancer Institute Common Terminology Criteria for Adverse Events [NCI-CTCAE] version 5.0, ≥grade 3);
2. Toxicities from previous antitumor therapy not yet resolved (NCI-CTCAE version 5.0, >grade 1); Unresolved skin reactions or hypersensitivity reactions of any etiology;
3. Untreated active brain metastases (including symptomatic brain metastases or leptomeningeal metastases); Subjects with treated brain metastases that are stable (stable lesions on brain imaging for ≥ 4 weeks prior to the first dose, no new neurological symptoms, or neurological symptoms returned to baseline, and no steroid therapy required for ≥ 14 days before first investigational treatment) may be enrolled;
4. History of malignancies other than breast cancer within 5 years prior to the first dose, except cured tumors such as carcinoma in situ, basal cell carcinoma, etc.;
5. Presence of any concurrent conditions including:
6. Severe or uncontrolled cardiovascular diseases: Such as Grade II or higher chronic congestive heart failure (New York Heart Association [NYHA] criteria), uncontrolled hypertension (excluding those stabilized with treatment where systolic blood pressure >150 mmHg and/or diastolic blood pressure >90 mmHg are controlled);
7. Definitive neurological disorders (e.g., epilepsy, dementia) or ≥grade 3 peripheral neuropathy;
8. Severe respiratory diseases requiring corticosteroid therapy (e.g., asthma, chronic obstructive pulmonary disease in acute exacerbation phase);
9. Uncontrolled diabetes (fasting blood glucose ≥ 10 mmol/L despite stable treatment);
10. Severe chronic or active infections requiring systemic antibacterial, antifungal or antiviral therapy (including tuberculosis) for which treatment has been administered for more than one week prior to the first dose and is expected to continue;
11. Active hepatitis B virus (HBV) (HBsAg-positive subjects must have HBV DNA ≤ ULN) or hepatitis C virus (HCV) infection [PCR-confirmed HCV RNA within normal range] or syphilis antibody-positive (confirmed) or HIV-positive;
12. Rash in any body area or conditions predisposing to skin reactions (e.g., cholestasis, systemic lupus erythematosus, eczema, allergic dermatitis, atopic dermatitis, herpes zoster, psoriasis);
13. History of any of the following:
14. Major surgery (e.g., abdominal, thoracic) within 28 days prior to first treatment dose, or anticipated need for major surgery during the study period;
15. Severe cardio/cerebrovascular events within 6 months prior to the first dose (asymptomatic lacunar infarcts allowed);
16. Subjects with any of the following prior/concurrent medication or treatment history
17. Subjects who have used corticosteroids, antihistamines, or other medications with therapeutic/preventive effects against skin rash within 2 weeks prior to first dose or within 5 half-lives (whichever is longer);
18. Subjects who have used small molecule chemotherapeutic agents (including endocrine therapy) within 2 weeks prior to first dose or 5 half-lives (whichever is longer), or received biological macromolecule antitumor therapy, biotherapy, radiotherapy, or require concurrent antitumor medications during the study period within 4 weeks prior to first dose;
19. Vaccination within 4 weeks prior to the first dose or anticipated vaccination during the study treatment period;
20. Subjects likely to undergo radiotherapy or hemodialysis within 2 months after first dose;
21. Subjects who are pregnant, breastfeeding, or planning pregnancy during the study;
22. Other circumstances where the investigator deems the subject unsuitable for participation in this study.

# Supplementary Table S1. Enrollment by study site and investigator

| **Site Name** | **Principal investigator** | **Approval ID** |
| --- | --- | --- |
| Harbin Medical University Cancer Hospital | Qingyuan Zhang | 23-218 |
| Liaoning Cancer Hospital & Institute | Tao Sun | 20230383 |
| The Fourth Hospital of Hebei Medical University (Hebei Tumor Hospital) | Cuizhi Geng | 202303 |
| The Second Affiliated Hospital of Anhui Medical University | Fanfan Li | YW2023-079 |
| Cancer Hospital of Xinjiang Medical University | Bing Zhao | [2023]伦审字号(024) |
| Shanxi Cancer Hospital | Guohui Han | YW2023040 |
| Tianjin Medical University Cancer Institute & Hospital | Zhongsheng Tong | E20230551 |
| Affiliated Hospital of Hebei University | Hua Yang | HDFYLL-GCP-2023-114 |
| Jiangsu Province Hospital, The First Affiliated Hospital with Nanjing Medical University | Yongmei Yin | 2023-MD-052 |
| Xingtai People’s Hospital | Xiangshun Kong | XTRM-23/0100 |
| Sun Yat-Sen Memorial Hospital, Sun Yat-Sen University | Ying Wang | SYSYW-2023-091-01 |
| Jilin Cancer Hospital | Lixia Ma | 202306-047-01 |
| Yunnan Tumor Hospital | Jianyun Nie | YW2023-023 |
| Beijing Hospital | Yongqiang Zhang | 2023BJYYEC-107-01 |
| Sichuan Academy of Medical Science & Sichuan Provincial People’s Hospital | Jing Luo | 伦审（药）2023年第56-1号 |
| Affiliated Hospital of Jining Medical University | Changping Shan | 2023-药-B024 |
| Union Hospital Tongji Medical College, Huazhong University of Science and Technology | Jing Yao | 2023伦审字(0269) |
| Guizhou Provincial People’s Hospital | Shisheng Tan | 伦审(药物)2023-18号 |
| The First Hospital of Lanzhou University | Xiaoling Ling | 2023伦审第(60)号 |
| Jiamusi Cancer and Tuberculosis Hospital | Hongmei Sun | 2023-伦审-10 |
| Cancer Hospital of Shandong First Medical University, Shandong Cancer Hospital | Huihui Li | SDZLEC2023-159-01 |
| Tengzhou Central Peoples Hospital | Kaixian Zhang | 2023-伦理审查-05-01 |
| Cangzhou Central Hospital | Guozhong Cui | 2023-119-01 |
| Qinghai University Affiliated Hospital | Jiuda Zhao | 2023-LLPJ-023 |
| Wuhu No.2 Peoples Hospital | Kewu Wang/  Yongming Wei | 2023-YW-001 |
| The Affiliated Cancer Hospital of Guizhou Medical University | Jianying Chang | FZ202303073 |
| Yuncheng Central Hospital | Zhaofeng Niu | 2023-YW-017 |
| Shaanxi Provincial People’s Hospital | Jianhui Li | (2023)伦审第(Y009)号 |
| Guangxi Medical University Cancer Hospital & Guangxi Cancer Institute | Weimin Xie | KS2024(393) |
| Daqing People’s Hospital | Shujuan Sun | 伦审意2023-013 |
| Sanmenxia Central Hospital | Ruiwen Zhang | IEC-C-008-A07-V2.0 |
| Affiliated Hospital of Hebei University of Engineering | Zhongchao Huo | 2023[Y]007 |
| Chongqing University Fuling Hospital | Qi Zhou | 2023-伦理审查-06 |
| The Third Xiangya Hospital of Central South University | Liyuan Qian | 23043 |
| Suining Central Hospital | Hongwei Yang | 202307 |
| The Affiliated Hospital of Southwest Medical University | Yunwei Han/  Lijia He | L2023016 |
| Xingyi People’s Hospital | Jiangping Feng | 2023001 |
| Anqing Municipal Hospital | Chenghui Li | (2023)药物伦审第(2)号 |
| The Second Affiliated Hospital of Guilin Medical University | Bihui Li | FYW2023008 |
| The First People’s Hospital of Nantong | Dongqin Chen | 2023-011-01 |
| Binzhou Medical University Hospital | Fangling Ning | 2023-030-01 |
| Gansu Provincial Hospital | Haiyun Huang | (药/械)伦审(2023)第(20)号 |
| The First Affiliated Hospital of Hainan Medical University | Yanda Lu | 2023(药物)第(69)号 |
| Chaoyang Central Hospital | Xiujie Cui | 医伦审[2023]3号 |
| Quzhou People’s Hospital | Qinhong Zheng | 2023-022-02 |
| First Affiliated Hospital of Gannan Medical University | Ruilian Xie | (2023)赣医伦审108号 |
| Wenzhou Central Hospital | Xiaoqing Hu | 温中心医伦审2023药第009号 |
| Northern Jiangsu People’s Hospital | Deyuan Fu | 2023017 |
| Fudan University affiliated Jinshan Hospital | Tiankui Qiao | JIEC 2023-E04 |
| Tangshan People’s Hospital | Xiaohong Wang | 伦理编号:2023-003 |
| Xiangyang Central Hospital | Tienan Yi | [2023]伦审字(023)号 |
| Yancheng No. 1 People’s Hospital | Ping Chen | [2023]伦审字(007) |
| The Second Affiliated Hospital of Soochow University | Zhixiang Zhuang | JD-LS2023017-I01 |
| Sichuan Tumor Hospital | Hao Wang | SCCHEC-01A-2023-043 |
| Shanghai Pudong Hospital | Minghua Yu | 2023-YW-006-F01 |
| Affiliated Hospital of Xuzhou Medical University | Zhengqiu Zhu | XYFY2023-YL033 |

# Supplementary Table S2. Demographic and clinical characteristics at baseline in ITT patients who received study drugs as first-line chemotherapy

| **Characteristics** | **SYHX2011**  **(*n* = 165)** | **PAB**  **(*n* = 167)** |
| --- | --- | --- |
| Age, years | | |
| Median (range) | 57.0 (35.0-80.0) | 56.0 (28.0-84.0) |
| <65, *n* (%) | 131 (79.4) | 135 (80.8) |
| ≥65, *n* (%) | 34 (20.6) | 32 (19.2) |
| Sex, *n* (%) | | |
| Male | 2 (1.2) | 0 (0.0) |
| Female | 163 (98.8) | 167 (100.0) |
| ECOG PS, *n* (%) | | |
| 0 | 63 (38.2) | 63 (37.7) |
| 1 | 102 (61.8) | 104 (62.3) |
| Menopause^a^, *n* (%) | | |
| Yes | 126 (76.4) | 123 (73.7) |
| No | 37 (22.7) | 44 (26.3) |
| Breast cancer molecular subtype, *n* (%) | | |
| HR-positive BC | 131 (79.4) | 126 (75.4) |
| TNBC | 34 (20.6) | 41 (24.6) |
| Clinical stage^b^, *n* (%) | | |
| Metastatic breast cancer (stage IV) | 165 (100.0) | 161 (96.4) |
| Stage IV at diagnosis | 97 (58.8) | 93 (55.7) |
| Relapsed or metastatic | 36 (21.8) | 45 (26.9) |
| Unknown | 32 (19.4) | 23 (13.8) |
| Unresectable, locally advanced breast cancer (stage IIIB and IIIC) | 0 (0.0) | 6 (3.6) |
| Metastatic organs, *n* (%) | | |
| Bone | 97 (58.8) | 102 (61.1) |
| Lymph nodes | 85 (51.5) | 81 (48.5) |
| Lung | 78 (47.3) | 69 (41.3) |
| Liver | 75 (45.5) | 76 (45.5) |
| Brain | 0 (0.0) | 6 (3.6) |
| Others | 53 (32.1) | 58 (34.7) |
| Prior taxane exposure and history of rash, *n* (%) | | |
| Prior taxanes with rash | 1 (0.6) | 1 (0.6) |
| Prior taxanes without rash | 100 (60.6) | 101 (60.5) |
| No prior taxanes | 64 (38.8) | 65 (38.9) |

^a^2 patients in the SYHX2011 group were male. ^b^TNM staging system. Abbreviations: BC, breast cancer; ECOG PS, Eastern Cooperative Oncology Group performance status; HR, hormone receptor; TNBC, triple negative breast cancer; ITT, intention to treat.

# Supplementary Table S3. Demographic and clinical characteristics at baseline in ITT patients who received study drugs as second or later-line chemotherapy

| **Characteristics** | **SYHX2011**  **(*n* = 64)** | **PAB**  **(*n* = 63)** |
| --- | --- | --- |
| Age, years | | |
| Median (range) | 55.5 (27.0-84.0) | 54.0 (28.0-72.0) |
| <65, *n* (%) | 52 (81.2) | 52 (82.5) |
| ≥65, *n* (%) | 12 (18.8) | 11 (17.5) |
| Sex, *n* (%) | | |
| Male | 0 (0.0) | 0 (0.0) |
| Female | 64 (100.0) | 63 (100.0) |
| ECOG PS, *n* (%) | | |
| 0 | 22 (34.4) | 32 (50.8) |
| 1 | 42 (65.6) | 31 (49.2) |
| Menopause, *n* (%) | | |
| Yes | 51 (79.7) | 49 (77.8) |
| No | 13 (20.3) | 14 (22.2) |
| Breast cancer molecular subtype, *n* (%) | | |
| HR positive BC | 42 (65.6) | 40 (63.5) |
| TNBC | 22 (34.4) | 23 (36.5) |
| Clinical stage^a^, *n* (%) | | |
| Metastatic breast cancer (stage IV) | 63 (98.4) | 62 (98.4) |
| Stage IV at diagnosis | 41 (64.1) | 33 (52.4) |
| Relapsed or metastatic | 10 (15.6) | 16 (25.4) |
| Unknown | 12 (18.8) | 13 (20.6) |
| Unresectable, locally advanced breast cancer (stage IIIB and IIIC) | 1 (1.6) | 1 (1.6) |
| Metastatic organs, *n* (%) | | |
| Bone | 39 (60.9) | 38 (60.3) |
| Lymph nodes | 32 (50.0) | 28 (44.4) |
| Lung | 30 (46.9) | 33 (52.4) |
| Liver | 30 (46.9) | 33 (52.4) |
| Brain | 3 (4.7) | 1 (1.6) |
| Others | 21 (32.8) | 17 (27.0) |
| Prior taxane exposure and history of rash, *n* (%) | | |
| Prior taxanes with rash | 2 (3.1) | 0 (0.0) |
| Prior taxanes without rash | 58 (90.6) | 58 (92.1) |
| No prior taxanes | 4 (6.2) | 5 (7.9) |

^a^TNM staging system. Abbreviations: BC, breast cancer; ECOG PS, Eastern Cooperative Oncology Group performance status; HR, hormone receptor; TNBC, triple negative breast cancer; ITT, intention to treat.

# Supplementary Table S4. Summary of dose reduction or interruption

| **Number of patients (%)** | **SYHX2011**  **(*n* = 228)** | **PAB**  **(*n* = 230)** |
| --- | --- | --- |
| At least one dose reduction or interruption | 74 (32.5) | 85 (37.0) |
| Reason for dose reduction or interruption^a^ | | |
| AEs | 49 (21.5) | 62 (27.0) |
| Others | 42 (18.4) | 39 (17.0) |
| Dose reduction | 27 (11.8) | 36 (15.7) |
| Number of dose reductions | | |
| 1 | 22 (9.6) | 29 (12.6) |
| 2 | 5 (2.2) | 7 (3.0) |
| Dose interruption | 63 (27.6) | 66 (28.7) |
| Infusion interruption | 0 (0.0) | 3 (1.3) |
| Dose delay | 63 (27.6) | 64 (27.8) |
| Number of dose delay | | |
| 1 | 45 (19.7) | 46 (20.0) |
| 2 | 13 (5.7) | 8 (3.5) |
| ≥3 | 5 (2.2) | 10 (4.3) |

^a^The summary of causes was calculated by frequency, as a patient may experience multiple interruptions with different underlying causes. Abbreviation: AEs, adverse events.

# Supplementary Table S5. Summary of efficacy assessed by IRC and investigator in patients who received study drugs as first-line chemotherapy (mITT and ITT)

| **Best overall response, *n* (%)** | **mITT** | | | | **ITT** | | | |
| --- | --- | --- | --- | --- | --- | --- | --- | --- |
|  | **IRC assessed** | | **Investigator assessed** | | **IRC assessed** | | **Investigator assessed** | |
|  | **SYHX2011**  **(*n* = 158)** | **PAB**  **(*n* = 159)** | **SYHX2011**  **(*n* = 158)** | **PAB**  **(*n* = 159)** | **SYHX2011**  **(*n* = 165)** | **PAB**  **(*n* = 167)** | **SYHX2011**  **(*n* = 165)** | **PAB**  **(*n* = 167)** |
| CR | 0 (0.0) | 1 (0.6) | 2 (1.3) | 2 (1.3) | 0 (0.0) | 1 (0.6) | 2 (1.2) | 2 (1.2) |
| PR | 62 (39.2) | 42 (26.4) | 65 (41.1) | 51 (32.1) | 62 (37.6) | 42 (25.1) | 65 (39.4) | 51 (30.5) |
| SD | 75 (47.5) | 88 (55.3) | 73 (46.2) | 80 (50.3) | 75 (45.5) | 88 (52.7) | 73 (44.2) | 80 (47.9) |
| PD | 17 (10.8) | 24 (15.1) | 16 (10.1) | 21 (13.2) | 17 (10.3) | 24 (14.4) | 16 (9.7) | 21 (12.6) |
| NE | 4 (2.5) | 4 (2.5) | 2 (1.3) | 5 (3.1) | 11 (6.7) | 12 (7.2) | 9 (5.5) | 13 (7.8) |
| ORR (95% CI), % | 39.2 (31.6-47.3) | 27.0 (20.3-34.7) | 42.4 (34.6-50.5) | 33.3 (26.1-41.2) | 37.6 (30.2-45.4) | 25.7 (19.3-33.1) | 40.6 (33.0-48.5) | 31.7 (24.8-39.4) |
| DCR (95% CI), % | 86.7 (80.4-91.6) | 82.4 (75.6-88.0) | 88.6 (82.6-93.1) | 83.6 (77.0-89.0) | 83.0 (76.4-88.4) | 78.4 (71.4-84.4) | 84.8 (78.5-89.9) | 79.6 (72.7-85.5) |

Abbreviations: CI, confidence interval; CR, complete response; DCR, disease control rate; IRC, independent review committee; ITT, intention to treat; mITT, modified intention to treat; NE, not evaluable; ORR, objective response rate; PD, progressive disease; PR, partial response; SD, stable disease.

# Supplementary Table S6. Summary of efficacy assessed by IRC and investigator in patients who received study drugs as second or later-line chemotherapy (mITT and ITT)

| **Best overall response, *n* (%)** | **mITT** | | | | **ITT** | | | |
| --- | --- | --- | --- | --- | --- | --- | --- | --- |
|  | **IRC assessed** | | **Investigator assessed** | | **IRC assessed** | | **Investigator assessed** | |
|  | **SYHX2011**  **(*n* = 57)** | **PAB**  **(*n* = 62)** | **SYHX2011**  **(*n* = 57)** | **PAB**  **(*n* = 62)** | **SYHX2011**  **(*n* = 64)** | **PAB**  **(*n* = 63)** | **SYHX2011**  **(*n* = 64)** | **PAB**  **(*n* = 63)** |
| CR | 0 (0.0) | 1 (1.6) | 1 (1.8) | 1 (1.6) | 0 (0.0) | 1 (1.6) | 1 (1.6) | 1 (1.6) |
| PR | 15 (26.3) | 13 (21.0) | 13 (22.8) | 8 (12.9) | 15 (23.4) | 13 (20.6) | 13 (20.3) | 8 (12.7) |
| SD | 31 (54.4) | 34 (54.8) | 31 (54.4) | 40 (64.5) | 31 (48.4) | 34 (54.0) | 31 (48.4) | 40 (63.5) |
| PD | 11 (19.3) | 13 (21.0) | 12 (21.1) | 13 (21.0) | 11 (17.2) | 13 (20.6) | 12 (18.8) | 13 (20.6) |
| NE | 0 (0.0) | 1 (1.6) | 0 (0.0) | 0 (0.0) | 7 (10.9) | 2 (3.2) | 7 (10.9) | 1 (1.6) |
| ORR (95% CI), % | 26.3 (15.5-39.7) | 22.6 (12.9-35.0) | 24.6 (14.1-37.8) | 14.5 (6.9-25.8) | 23.4 (13.8-35.7) | 22.2 (12.7-34.5) | 21.9 (12.5-34.0) | 14.3 (6.7-25.4) |
| DCR (95% CI), % | 80.7 (68.1-90.0) | 77.4 (65.0-87.1) | 78.9 (66.1-88.6) | 79.0 (66.8-88.3) | 71.9 (59.2-82.4) | 76.2 (63.8-86.0) | 70.3 (57.6-81.1) | 77.8 (65.5-87.3) |

Abbreviations: CI, confidence interval; CR, complete response; DCR, disease control rate; IRC, independent review committee; ITT, intention to treat; mITT, modified intention to treat; NE, not evaluable; ORR, objective response rate; PD, progressive disease; PR, partial response; SD, stable disease.

# Supplementary Table S7. Summary of serious TEAEs and TRAEs.

| **Incidence, *n* (%)** | **SYHX2011 group**  **(*n* = 228)** | **PAB group**  **(*n* = 230)** |
| --- | --- | --- |
| **Serious TEAEs** | 28 (12.3) | 40 (17.4) |
| **Common serious TEAEs^a^** |  |  |
| AST increased | 3 (1.3) | 3 (1.3) |
| Pleural effusion | 3 (1.3) | 1 (0.4) |
| ALT increased | 2 (0.9) | 4 (1.7) |
| Infective pneumonia | 2 (0.9) | 4 (1.7) |
| Anemia | 2 (0.9) | 3 (1.3) |
| Peripheral sensory neuropathy | 2 (0.9) | 3 (1.3) |
| Neutropenia | 2 (0.9) | 1 (0.4) |
| Leukopenia | 1 (0.4) | 2 (0.9) |
| Cerebral infarction | 1 (0.4) | 2 (0.9) |
| Thrombocytopenia | 0 (0.0) | 3 (1.3) |
| Febrile neutropenia | 0 (0.0) | 3 (1.3) |
| Dyspnea | 0 (0.0) | 2 (0.9) |
| Asthenia | 0 (0.0) | 2 (0.9) |
| Diarrhea | 0 (0.0) | 2 (0.9) |
| **Serious TRAEs** | 21 (9.2) | 28 (12.2) |
| **Common serious TRAEs^b^** |  |  |
| AST increased | 3 (1.3) | 3 (1.3) |
| ALT increased | 2 (0.9) | 4 (1.7) |
| Peripheral sensory neuropathy | 2 (0.9) | 3 (1.3) |
| Anemia | 2 (0.9) | 3 (1.3) |
| Infective pneumonia | 2 (0.9) | 1 (0.4) |
| Neutropenia | 2 (0.9) | 1 (0.4) |
| Leukopenia | 1 (0.4) | 2 (0.9) |
| Thrombocytopenia | 0 (0.0) | 3 (1.3) |
| Febrile neutropenia | 0 (0.0) | 3 (1.3) |
| Diarrhea | 0 (0.0) | 2 (0.9) |
| Asthenia | 0 (0.0) | 2 (0.9) |

^a^Common serious TEAEs is defined as TEAEs that occurring in ≥2 patients in either group. ^b^Common serious TRAEs is defined as TRAEs that occurring in ≥2 patients in either group. Abbreviations: ALT, alanine aminotransferase; AST, aspartate aminotransferase; TEAEs, treatment emergent adverse events; TRAEs, treatment related adverse events.

# Supplementary Table S8. Summary of TEAEs and TRAEs leading to permanent treatment discontinuation, interruption and dose reduction.

| **Incidence, *n* (%)** | **SYHX2011 group**  **(*n* = 228)** | **PAB group**  **(*n* = 230)** |
| --- | --- | --- |
| **TEAEs leading to treatment discontinuation** | 5 (2.2) | 16 (7.0) |
| **TRAEs leading to treatment discontinuation** | 5 (2.2) | 15 (6.5) |
| Common TRAEs leading to treatment discontinuation^a^ |  |  |
| Peripheral sensory neuropathy | 3 (1.3) | 4 (1.7) |
| Peripheral neuropathy | 0 (0.0) | 3 (1.3) |
| **TEAEs leading to dose reduction** | 28 (12.3) | 37 (16.1) |
| **TRAEs leading to dose reduction**^b^ | 28 (12.3) | 36 (15.7) |
| Common TRAEs leading to dose reduction^c^ |  |  |
| Peripheral sensory neuropathy | 16 (7.0) | 17 (7.4) |
| Asthenia | 3 (1.3) | 3 (1.3) |
| Leukopenia | 2 (0.9) | 2 (0.9) |
| Neutropenia | 2 (0.9) | 1 (0.4) |
| Hypoesthesia | 2 (0.9) | 1 (0.4) |
| Anemia | 2 (0.9) | 1 (0.4) |
| Peripheral neuropathy | 1 (0.4) | 4 (1.7) |
| Neurotoxicity | 0 (0.0) | 3 (1.3) |
| Thrombocytopenia | 0 (0.0) | 3 (1.3) |
| Peripheral motor neuropathy | 0 (0.0) | 2 (0.9) |
| Blood bilirubin elevated | 0 (0.0) | 2 (0.9) |
| **TEAEs leading to treatment interruption** | 54 (23.7) | 56 (24.3) |
| **TRAEs leading to treatment interruption** | 46 (20.2) | 41 (17.8) |
| Common TRAEs leading to treatment interruption^d^ |  |  |
| Peripheral sensory neuropathy | 9 (3.9) | 9 (3.9) |
| Peripheral neuropathy | 6 (2.6) | 3 (1.3) |
| ALT increased | 5 (2.2) | 6 (2.6) |
| Neutropenia | 5 (2.2) | 3 (1.3) |
| AST increased | 4 (1.8) | 4 (1.7) |
| Asthenia | 4 (1.8) | 4 (1.7) |
| Upper respiratory infection | 3 (1.3) | 2 (0.9) |
| Leukopenia | 2 (0.9) | 2 (0.9) |
| Thrombocytopenia | 2 (0.9) | 2 (0.9) |
| γ-GGT elevation | 2 (0.9) | 1 (0.4) |
| Anemia | 2 (0.9) | 1 (0.4) |
| Appetite decreased | 1 (0.4) | 2 (0.9) |
| LDH increased | 0 (0.0) | 2 (0.9) |
| Fever | 0 (0.0) | 2 (0.9) |
| Cough | 0 (0.0) | 2 (0.9) |
| **TEAEs leading to death** | 4 (1.8) | 3 (1.3) |
| **TRAEs leading to death** | 3 (1.3) | 0 (0.0) |
| Infective pneumonia | 1 (0.4) | 0 (0.0) |
| Infectious shock | 1 (0.4) | 0 (0.0) |
| Sepsis | 1 (0.4) | 0 (0.0) |

^a^Common TRAEs leading to treatment discontinuation is defined as TRAEs that occurring in ≥1% of patients in either group. ^b^A patient may experience multiple adverse events leading to dose reduction. ^c^Common TRAEs leading to dose reduction is defined as TRAEs that occurring in ≥2 patients in either group. ^d^Common TRAEs leading to treatment interruption is defined as TRAEs that occurring in ≥2 patients in either group. ALT, alanine aminotransferase; AST, aspartate aminotransferase; GGT, glutamyl transferase; LDH, Lactate Dehydrogenase; TEAEs, treatment emergent adverse events; TRAEs, treatment related adverse events.


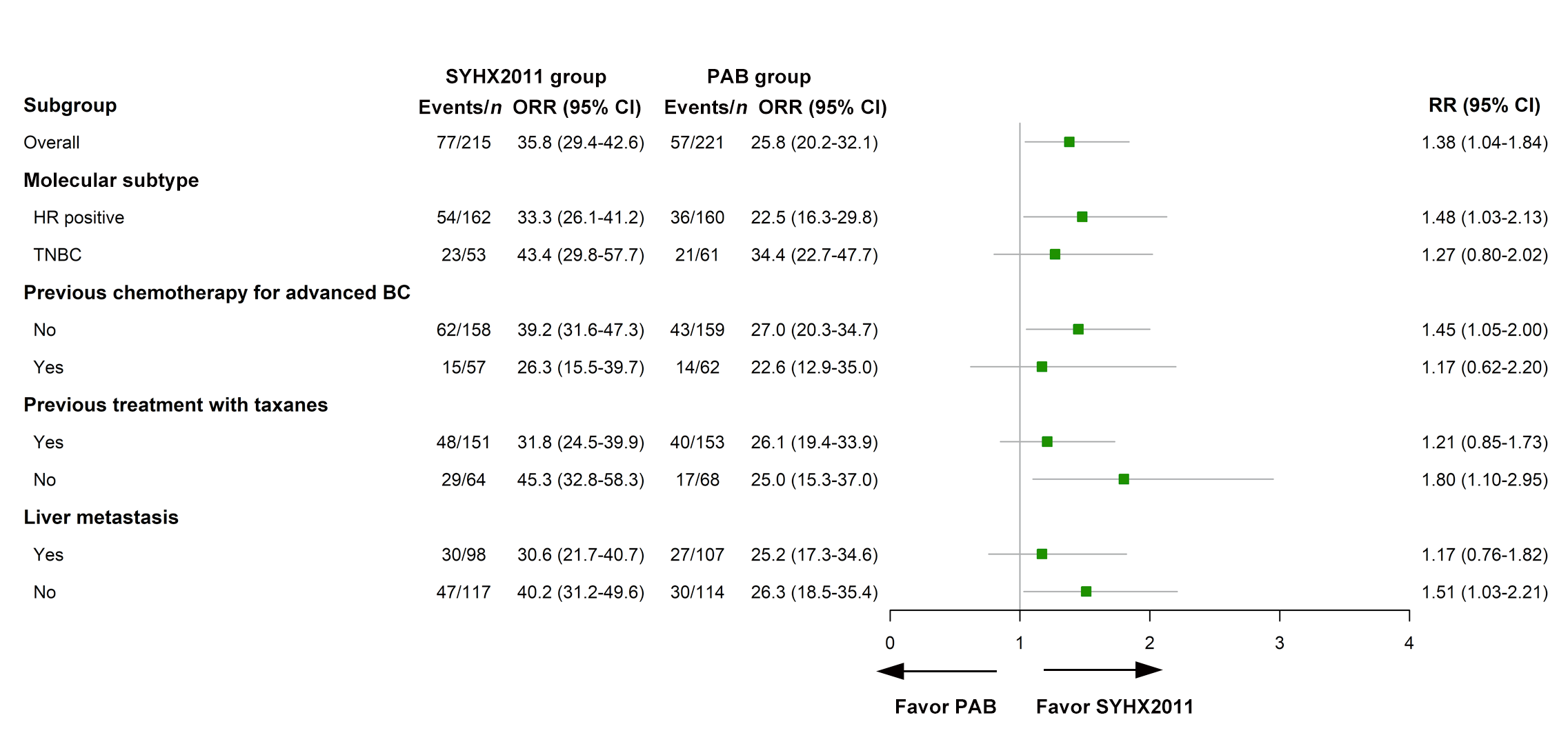


# Supplementary Figure S1. Subgroup analysis of objective response rate assessed by IRC in the mITT population. Abbreviations: CI, confidence interval; HR, hormone receptor; IRC, independent review committee; mITT, modified intention to treat; ORR, objective response rate; RR, rate ratio; TNBC, triple negative breast cancer.


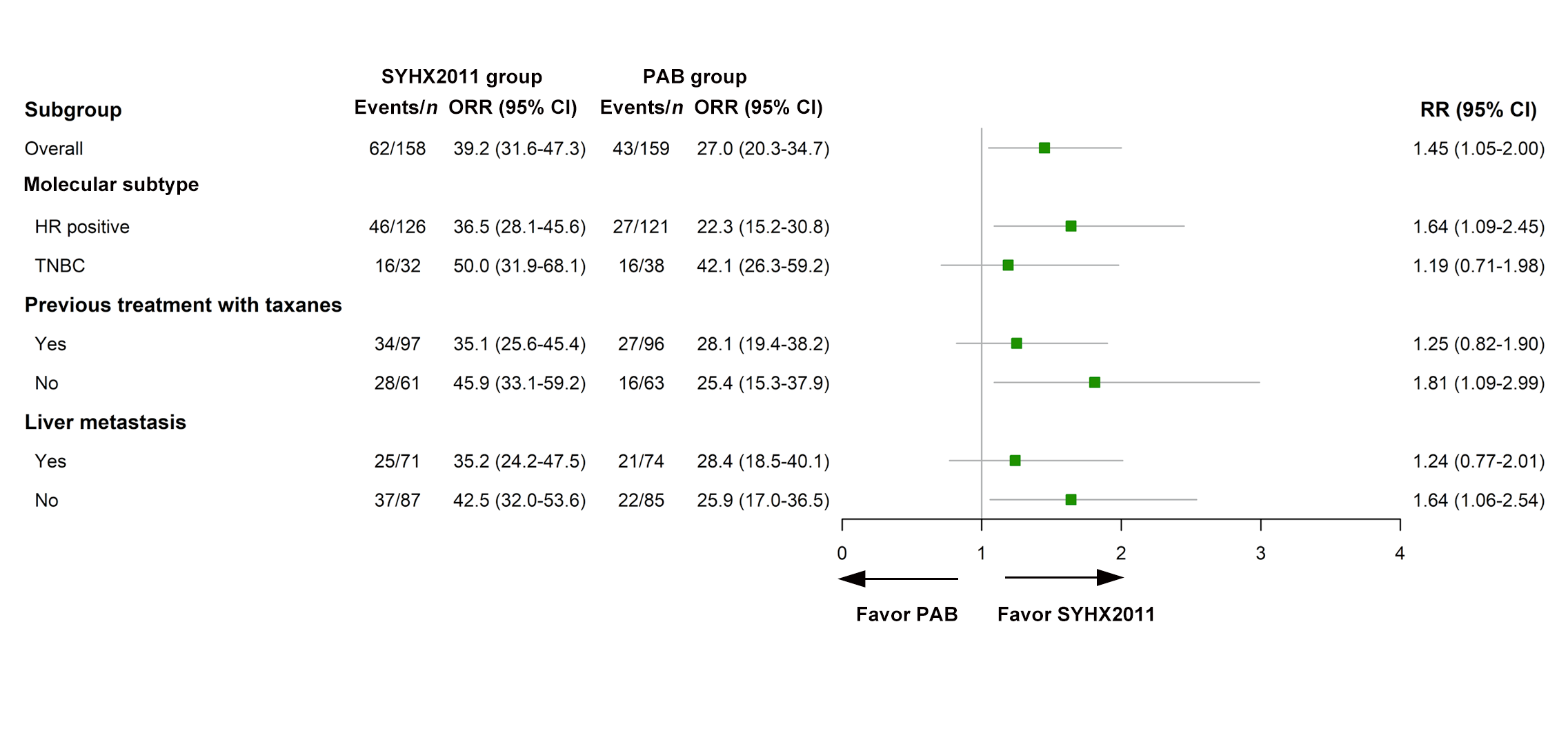


# Supplementary Figure S2. Subgroup analysis of objective response rate assessed by IRC in mITT patients who received study drugs as first-line chemotherapy. Abbreviations: CI, confidence interval; HR, hormone receptor; IRC, independent review committee; mITT, modified intention to treat; ORR, objective response rate; RR, rate ratio; TNBC, triple negative breast cancer.

#
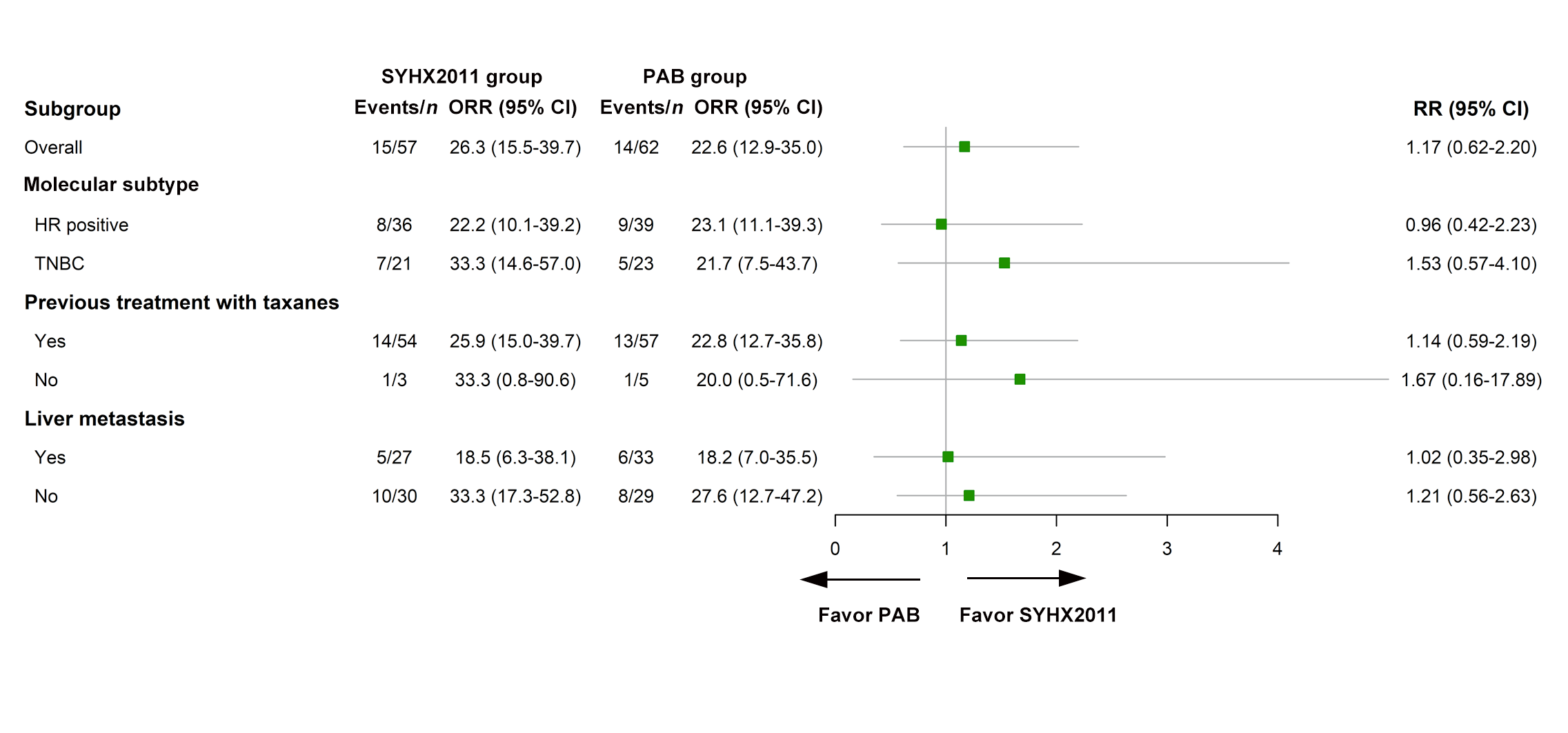
Supplementary Figure S3. Subgroup analysis of objective response rate assessed by IRC in mITT patients who received study drugs as second or later-line chemotherapy. Abbreviations: CI, confidence interval; HR, hormone receptor; IRC, independent review committee; mITT, modified intention to treat; ORR, objective response rate; RR, rate ratio; TNBC, triple negative breast cancer.


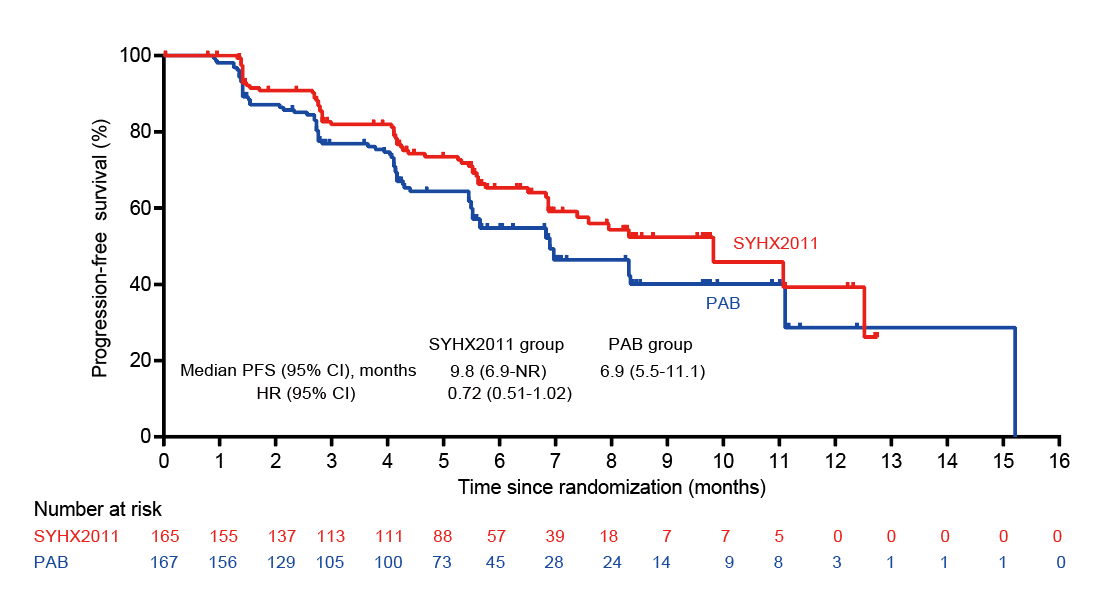


# Supplementary Figure S4. Kaplan-Meier curve of progression-free survival assessed by IRC in ITT patients who received study drugs as first-line chemotherapy. Abbreviations: CI, confidence interval; IRC, independent review committee; ITT, intention to treat; NR, not reached; PFS, progression-free survival.


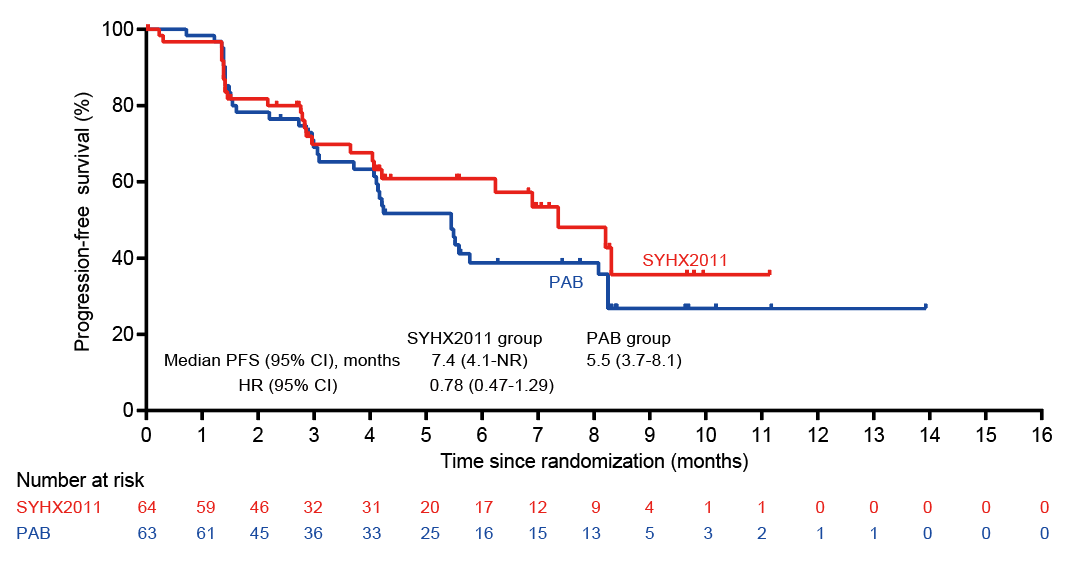


# Supplementary Figure S5. Kaplan-Meier curve of progression-free survival assessed by IRC in ITT patients who received study drugs as second or later-line chemotherapy. Abbreviations: CI, confidence interval; IRC, independent review committee; ITT, intention to treat; NR, not reached; PFS, progression-free survival.


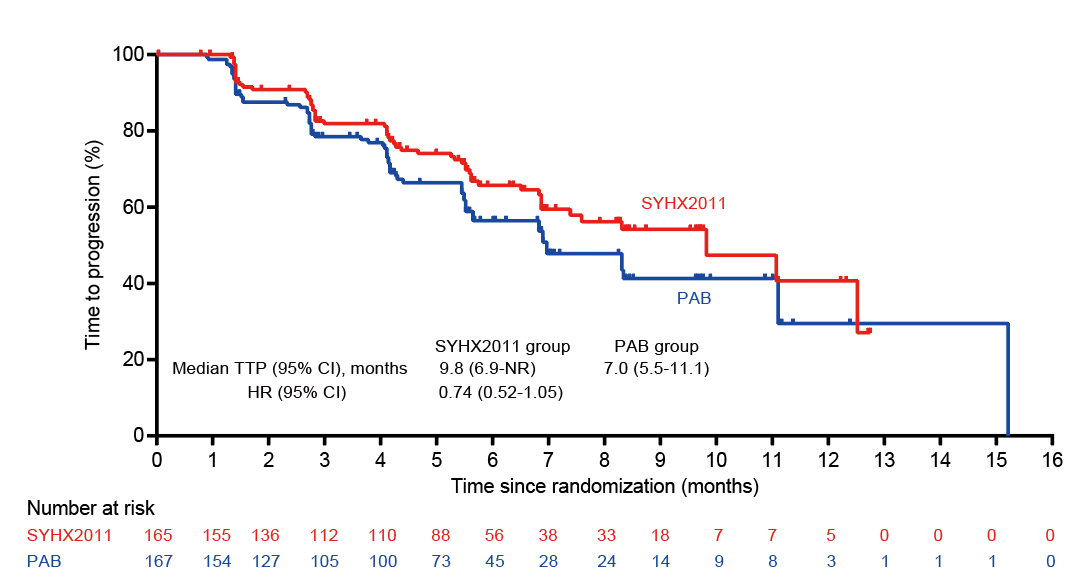


# Supplementary Figure S6. Kaplan-Meier curve of time to progression assessed by IRC in ITT patients who received study drugs as first-line chemotherapy. Abbreviations: CI, confidence interval; IRC, independent review committee; ITT, intention to treat; NR, not reached; TTP, time to progression.


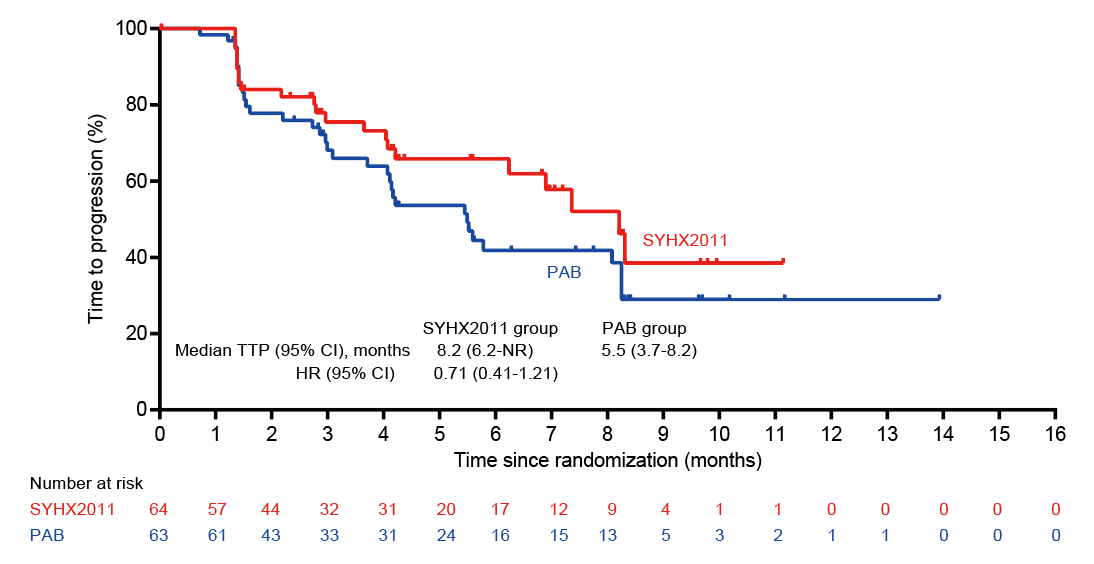


# Supplementary Figure S7. Kaplan-Meier curve of time to progression assessed by IRC in ITT patients who received study drugs as second or later-line chemotherapy. Abbreviations: CI, confidence interval; IRC, independent review committee; ITT, intention to treat; NR, not reached; TTP, time to progression.


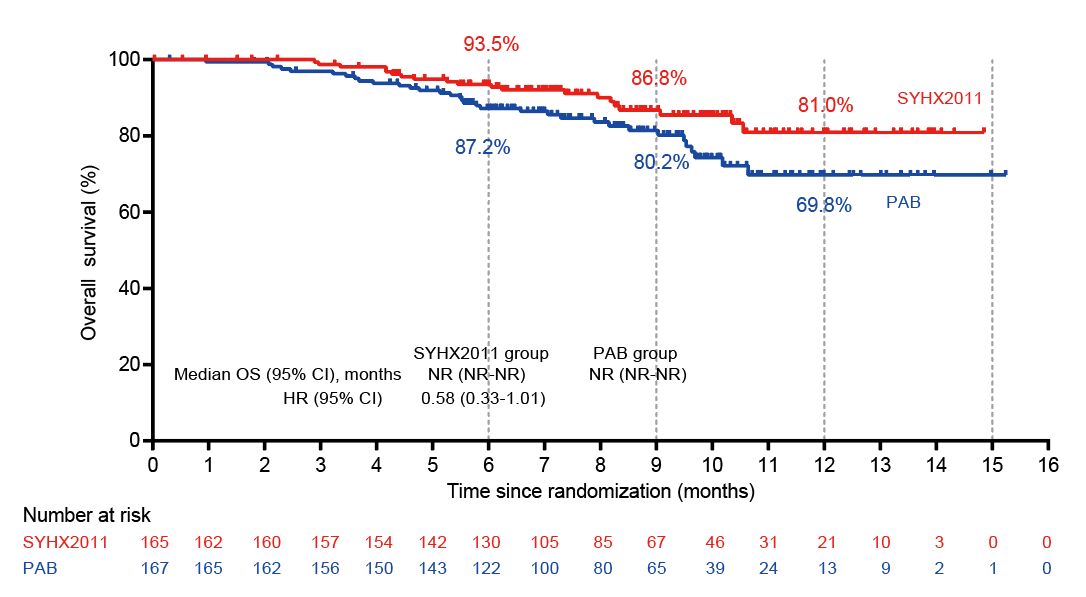


# Supplementary Figure S8. Kaplan-Meier curve of overall survival in ITT patients who received study drugs as first-line chemotherapy. Abbreviations: CI, confidence interval; ITT, intention to treat; NR, not reached; OS, overall survival.


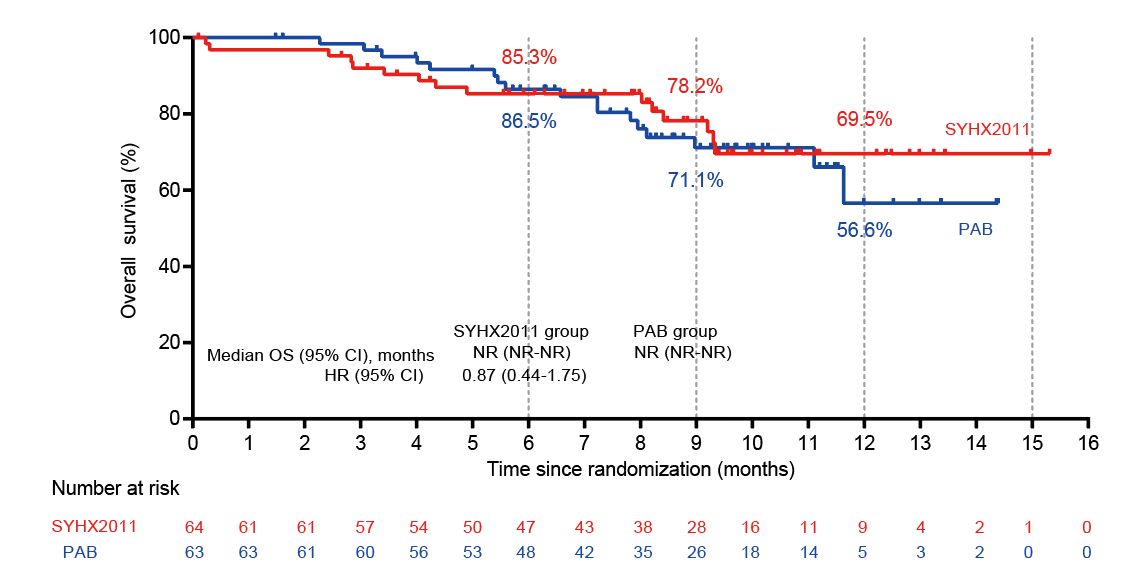


# Supplementary Figure S9. Kaplan-Meier curve of overall survival in ITT patients who received study drugs as second or later-line chemotherapy. Abbreviations: CI, confidence interval; ITT, intention to treat; NR, not reached; OS, overall survival.
